# Characterization of a MOB1 Homolog in the Apicomplexan Parasite *Toxoplasma gondii*

**DOI:** 10.3390/biology10121233

**Published:** 2021-11-26

**Authors:** Inês L. S. Delgado, Alexandra Tavares, Samuel Francisco, Dulce Santos, João Coelho, Afonso P. Basto, Sara Zúquete, Joachim Müller, Andrew Hemphill, Markus Meissner, Helena Soares, Alexandre Leitão, Sofia Nolasco

**Affiliations:** 1 CIISA—Centro de Investigação Interdisciplinar em Sanidade Animal, Faculdade de Medicina Veterinária, Universidade de Lisboa, 1300-477 Lisboa, Portugal or ines.delgado@ulusofona.pt (I.L.S.D.); ajtavares@igc.gulbenkian.pt (A.T.); adsfrancisco@fmv.ulisboa.pt (S.F.); dulcesantos@fmv.ulisboa.pt (D.S.); jnscoelho@gmail.com (J.C.); abasto@fmv.ulisboa.pt (A.P.B.); s.zuquete@edu.ulisboa.pt (S.Z.); alexandre@fmv.ulisboa.pt (A.L.); 2 Faculdade de Medicina Veterinária, Universidade Lusófona, 1749-024 Lisboa, Portugal; 3 Institute of Parasitology, Department of Infectious Diseases and Pathobiology, Vetsuisse Faculty, University of Bern, Länggass-Strasse 122, CH-3012 Bern, Switzerland; joachim.mueller@vetsuisse.unibe.ch (J.M.); andrew.hemphill@vetsuisse.unibe.ch (A.H.); 4 Institute for Experimental Parasitology, Faculty of Veterinary Medicine, Ludwig-Maximilians-Universität Munich, D-82152 Munich, Germany; Markus.Meissner@para.vetmed.uni-muenchen.de; 5 Escola Superior de Tecnologia da Saúde de Lisboa, Instituto Politécnico de Lisboa, 1990-096 Lisboa, Portugal; mhsoares@fc.ul.pt or; 6 Centro de Química Estrutural–Faculdade de Ciências da Universidade de Lisboa, 1749-016 Lisboa, Portugal

**Keywords:** *Toxoplasma gondii*, tachyzoite, MOB1, Hippo pathway, mitotic exit network, apicomplexa

## Abstract

**Simple Summary:**

Monopolar spindle One Binder1 (MOB1) proteins regulate key cellular functions, namely cell multiplication and cell division. The unicellular parasite *Toxoplasma gondii* transitions between several morphological stages, with the need to control the number of parasites in its cellular environment. We hypothesized that MOB1 proteins could participate in the regulation of the *T. gondii* life cycle, having identified one MOB1 protein (TgMOB1) coded in its genome. However, this study shows that TgMOB1 presents divergent features. While in organisms studied to date the lack of MOB1 has led to cell division defects, this did not occur in *T. gondii* in vitro cultures where *mob1* was not an essential gene. Additionally, the identification of TgMOB1 proximity interacting partners detected novel MOB1 interactors. Still, TgMOB1 localizes to the region between the new-forming nuclei during cell division, and *T. gondii* parasites multiply slower with TgMOB1 overexpression and faster when there is a lack of TgMOB1, indicating an intricate role for TgMOB1 in *T. gondii*. This study uncovers new features of the *T. gondii* biology, a zoonotic parasite and model organism for the phylum Apicomplexa, and highlights the complex roles MOB1 proteins may assume, with possible implications for disease processes.

**Abstract:**

Monopolar spindle One Binder1 (MOB1) proteins are conserved components of the tumor-suppressing Hippo pathway, regulating cellular processes such as cytokinesis. Apicomplexan parasites present a life cycle that relies on the parasites’ ability to differentiate between stages and regulate their proliferation; thus, Hippo signaling pathways could play an important role in the regulation of the apicomplexan life cycle. Here, we report the identification of one MOB1 protein in the apicomplexan *Toxoplasma gondii*. To characterize the function of MOB1, we generated gain-of-function transgenic lines with a ligand-controlled destabilization domain, and loss-of-function clonal lines obtained through CRISPR/Cas9 technology. Contrary to what has been characterized in other eukaryotes, MOB1 is not essential for cytokinesis in *T. gondii*. However, this picture is complex since we found MOB1 localized between the newly individualized daughter nuclei at the end of mitosis. Moreover, we detected a significant delay in the replication of overexpressing tachyzoites, contrasting with increased replication rates in knockout tachyzoites. Finally, using the proximity-biotinylation method, BioID, we identified novel members of the MOB1 interactome, a probable consequence of the observed lack of conservation of some key amino acid residues. Altogether, the results point to a complex evolutionary history of MOB1 roles in apicomplexans, sharing properties with other eukaryotes but also with divergent features, possibly associated with their complex life cycle.

## 1. Introduction

*Toxoplasma gondii* is a unicellular parasitic eukaryotic organism that presents a highly polarized cell and an obligatory intracellular lifestyle during most of its life cycle. It is often considered the world’s most successful parasite as it is believed to infect all warm-blooded animals with frequently high prevalence rates. Part of the secret to its success is its ability to remain viable and mostly undetected in the host through regulatory mechanisms of proliferation and parasite number control. In the asexual life cycle phase, the parasites disseminate to different intermediate host tissues as rapidly proliferating tachyzoites that replicate by endodyogeny inside intracellular parasitophorous vacuoles [1]. Upon the onset of immunity, tachyzoites convert to slowly replicating bradyzoites that form tissue cysts, which may remain viable through the lifetime of the host [1]. Sexual reproduction of *T. gondii* takes place in the intestinal epithelium of felids, first going through schizogony driven asexual expansion, followed by gamogony, fecundation, and oocyst excretion into the environment, where two sporocysts containing eight sporozoites remain latent inside the very resistant oocyst wall [2]. Therefore, *T. gondii* presents a complex life cycle that relies on its ability to differentiate between several stages and regulate its proliferation.

Monopolar spindle One Binder (MOB) proteins form a conserved eukaryote family of kinase adaptors that regulate key cellular processes, namely chromosome segregation, mitotic exit, and cytokinesis [3,4,5,6,7,8]. MOB proteins also regulate cell polarity, contributing to the definition of cell morphology and accurate cell division, and are frequently present in structures that are fundamental for these processes, namely the spindle pole body and the centrosome [8,9,10,11]. The function of MOB proteins in cell division seems to be deeply conserved throughout the eukaryotic tree of life [4,5,7,8,12,13]. These proteins are pivotal members of the yeast mitotic exit network (MEN)/septation initiation network (SIN) and the multicellular eukaryote Hippo signaling pathway, where upstream signals lead to the activation of the core kinase module through a Cdc15p/MST1/2 kinase that in turn activates a Dbf2p/LATS1/2 kinase, enhanced by the MOB1 kinase adaptor [14,15,16,17]. MEN/SIN activation results in mitotic exit and cytokinesis through the effector proteins Cdc14p/Cip1p and Cdk1p, while Hippo signaling activation induces inhibition of pro-mitotic and anti-apoptotic associated transcription. Among the proteins that constitute the Hippo signaling pathway, MOB1 is the one that appears earlier in eukaryotes, supporting a core role for this protein [18]. Most eukaryotes, from budding yeast to humans, present more than one MOB protein [19]. This is also the case in *Trypanosoma brucei*—the only parasitic organism in which MOB1 proteins have been investigated to date—which possesses MOB1A and MOB1B proteins and in which MOB1 depletion leads to cytokinesis failure [5].

A large-scope review and functional studies have analyzed the presence and function of MOB1 proteins across the tree of life [18,20,21]. Still, no published studies have analyzed the functional role of MOB1 proteins in members of the phylum Apicomplexa, for which *T. gondii* is regarded as the prime model organism. *T. gondii* presents one gene encoding for a MOB1 protein (TgMOB1). Data sets accessible in ToxoDB (www.toxodb.org accessed on 20 October 2021, [22,23,24]) suggest that the *mob1* gene expression in tachyzoites is at low levels. However, a functional characterization of this protein is lacking and is certainly relevant to better understand the replication of *T. gondii* and, potentially, to develop live, attenuated anti-*T. gondii* vaccines. In order to fill this gap, we generated clonal *T. gondii* cell lines for the study of *mob1* gain-of-function, through the random integration of *mob1*-tagged constructs, and of *mob1* loss-of-function using CRISPR/Cas9 technology adapted to *T. gondii* [25]. We also obtained *T. gondii* cell lines expressing endogenously tagged TgMOB1 to determine its subcellular localization [26] and identified the TgMOB1 interactome using the proximity-dependent biotin labeling method, BioID [27].

## 2. Materials and Methods

### 2.1. Deduced Amino Acid Sequence Analysis

Multiple sequence alignments of previously studied MOB1 proteins and of apicomplexan MOB1 proteins were performed with the CLUSTAL W Multiple Sequence Alignment Program (version 1.83, February 2003, available at genome.jp/tools-bin/clustalw accessed on 5 May 2021) using the default settings, BLOSUM weight matrix for protein with gap open penalty of 10 and gap extension penalty of 0.5. Sequence similarities and identities were calculated with SIAS (available at imed.med.ucm.es/Tools/sias.html accessed on 5 May 2021) using the default parameters, namely sequence length determined by the length of the smallest sequence and BLOSUM62 weight matrix with gap open penalty of 10 and gap extension penalty of 0.5. The SIAS webpage divides amino acids into the following similarity groups: aromatic (F, Y, and W), aliphatic (I, L, and V), positively charged (H, K, and R), negatively charged (D and E), small with a hydroxyl group (S and T), and neutral polar (N and Q). The five remaining amino acids (A, C, G, M, and P) are not included in any similarity groups. For a graphical representation, the ClustalW alignment was loaded into the Sequence Manipulation Suite Color Align Conservation tool using as a setting the minimum percentage of agreeing sequences for identity or similarity shading to 70% and considering the default amino acid similarity groups (GAVLI, FYW, CM, ST, KRH, DENQ, P) [28]. *T. gondii* MOB1 secondary and theoretical 3D structures were obtained from the SWISS-MODEL interface [29,30,31]. *Saccharomyces cerevisiae* ScMOB1 (Mob1p; NP_012160.2) protein secondary structures were obtained from the SWISS-MODEL interface, while interaction motifs were obtained from Mrkobrada et al. [32] and Parker et al. [33]. Sequence accession numbers are shown in Table S1. Whenever multiple MOB1 isotypes existed, the MOB1A isotype was used for the analysis.

### 2.2. Phylogenetic Analysis

The MOB1 protein sequences of the different organisms used in the phylogenetic analysis were obtained from BLAST, using *Homo sapiens* HsMOB1 and *S. cerevisiae* ScMOB1. Protein–protein BLAST searches (with a threshold of 0.0001 and remaining options as default) were performed against the UniProtKB database. The MOB2 protein sequences of *Danio rerio*, *Mus musculus*, and *H. sapiens* were used as an out group. Sequence accession numbers are shown in Table S1. Whenever multiple MOB1 isotypes existed, the MOB1A isotype was used for the analysis. Protein sequence alignments were performed using M-COFFEE [34] and curated with TrimAl [35], both with default settings. An analysis of the alignment and a comparison of sequences was performed using Jalview [36] and Base-By-Base [37]. ProtTest 3.0 [38] was used to select the best model for phylogenetic tree construction. Maximum likelihood (ML) trees with 1,000 bootstrap replicates were constructed using a combination of PhyML [39], with settings indicated by previous determination of the best phylogenetic model, and the tools seqboot, consense, and retree from the PHYLIP package version 3.695. Trees were edited using MEGA 6 [40].

### 2.3. DNA Constructs

Total RNA was extracted from freshly egressed RH tachyzoites using the E.Z.N.A. Total RNA Kit I (Omega Bio-Tek, Inc., Norcross, GA, USA), following the manufacturer’s instructions. Genomic DNA contamination was eliminated with RNase-free DNase Set I (Omega Bio-Tek, Inc., Norcross, GA, USA). Complimentary DNA (cDNA) was synthesized using SuperScript III Reverse Transcriptase (Invitrogen; Thermo Fisher Scientific, Inc., Waltham, MA, USA), following the manufacturer’s instructions. The cDNA synthesis was performed using 1 μg of total RNA and Oligo (dT) primer (Invitrogen; Thermo Fisher Scientific, Inc., Waltham, MA, USA). The open reading frame coding for TgMOB1 (TGME49_104730) was amplified from cDNA by PCR with specific primers containing restriction sites compatible with the target plasmids whenever appropriate. The primers used to obtain the DNA constructs are described in Table S2.

To obtain a strain that overexpresses TgMOB1 at controlled levels, the *mob1* cDNA was cloned in fusion with the N-terminal of the egfp coding sequence into the mammalian pIC111 vector using the *Bam*HI and *Eco*RV restriction sites. The *mob1*-*gfp* coding sequence was then sub-cloned into the *T. gondii p5RT70-ddfkbp-my-hx* plasmid using the *Not*I and *Bam*HI restriction sites, producing the plasmid *p5RT70-ddfkbp-myc-mob1-gfp-hx*. The *5RT70* promoter sequence is a strong promoter from tubulin, leading to high overexpression levels. The expression of the fused protein is controlled by the protein destabilization domain (dd), ddFKBP, which allows for the control of protein expression levels using the Shield1 ligand (Shd; Clontech, Takara Bio, Inc., Kusatsu, Japan) which binds to the dd [41]. In the absence of Shd, MOB1-GFP is labeled and degraded by the proteasome, while in the presence of Shd, the MOB1-GFP protein is protected.

In order to employ the BioID method, we obtained BirA*-expressing clonal lines. *Mob1* was cloned in fusion with the C-terminal of the birA* coding sequence into the mammalian pcDNA5-flag-birA* vector using the *Xho*I and *Not*I restriction sites. The *T. gondii* vector *p5RT70-loxP-myc-loxP-yfp-hx* was linearized with *Afl*II and *Nde*I, which simultaneously removed the *loxP-yfp* fragment. The flag-birA* and flag-birA*-mob1 fragments were amplified from pcDNA5-flag-birA* and pcDNA5-flag-birA*-mob1, respectively, and were inserted into the hydrolyzed vector using the *Afl*II and *Nde*I restriction sites, resulting in the p5RT70-loxP-myc-flag-birA*-hx and p5RT70-loxP-myc-flag-birA*-mob1-hx vectors.

To obtain a strain overexpressing TgMOB1 at low levels, *mob1* was cloned into the *pmorn1-morn1-myc-cat* vector through ligation-independent cloning (LIC) using the In-Fusion HD Cloning Kit (Takara Bio, Inc., Kusatsu, Japan), following the manufacturer’s instructions. MORN1 is a protein constitutively expressed at low levels, localized most notably at the basal complex [42]. The *morn1* promoter was used as a moderate expression promoter for *mob1*. The *pmorn1-morn1-myc-cat* vector was linearized using the *Bgl*II and *Asc*I restriction endonucleases which simultaneously removed the *morn1-myc* fragment. The *mob1* fragment was amplified from the *p5RT70-dd-myc-mob1-gfp-hx* vector and first cloned in the pJET1.2/blunt cloning vector (Thermo Fisher Scientific, Inc., Waltham, MA, USA) in order to fuse the flag and *mob1* sequences (the flag sequence was inserted in the forward primer). From the pJET1.2/blunt-*flag-mob1* vector, the *flag-mob1* fragment was amplified with the LIC primers. Finally, the *flag-mob1* fragment was exchanged with the *morn1-myc* fragment, obtaining the *pmorn1-flag-mob1-cat* vector using the In-Fusion HD Cloning Kit protocol (Takara Bio, Inc., Kusatsu, Japan).

We applied the endogenous tagging technique described by Huynh and Carruthers [26] to obtain a strain expressing the endogenous *mob1* fused to a tag. A 2 Kb region of the C-terminal end of the *mob1* genomic locus was amplified (excluding the nucleotides coding for the STOP codon) and cloned into the *pLIC-yfp-dhfr* (LIC—ligation-independent cloning site) plasmid vector in frame with the *yfp* coding sequence, producing the *pLIC-mob1-yfp-dhfr* vector.

To obtain a *mob1* functional knockout, the cloning of *mob1* specific gRNAs in the *T. gondii pU6-sgRNA-dhfr* vector was achieved through direct mutagenesis using the Q5 Site-Directed Mutagenesis Kit (New England Biolabs, Inc., Ipswich, MA, USA), following the manufacturer’s instructions. The gRNAs were designed with the Eukaryotic Pathogen CRISPR sgRNA Design Tool (EuPaGDT) using the *T. gondii* GT1 ToxoDB-26 release, the most fully annotated genome available at the time closest to the RH genome [43]. The design of the sgRNAs took into account the specificities of the Cas9, namely, sequences with 20 nucleotides figuring immediately before a 5′-NGG-3′ protospacer-adjacent motif (PAM). Each gRNA was cloned independently into the vector *pU6-sgRNA-dhfr* in order to form an sgRNA by fusion with the trRNA sequence included in the plasmid. The resulting plasmid constructs (*pU6-sgRNA1-dhfr*, *pU6-sgRNA2-dhfr*, and *pU6-sgRNA3-dhfr*) were transfected independently into RHCas9 tachyzoites.

### 2.4. Toxoplasma Gondii Strains, Culture, and Transfection

We analyzed the type I strain RHΔHx, referred to here as RH [44], maintained by sequential passages through in vitro culture, and used it to obtain MOB1 recombinant protein-expressing strains. We also employed two modified RH strains: the RHΔku80 strain, to obtain a *mob1* C-terminal tagged strain as it deleted for the ku80 gene, which favors the homologous recombination DNA repair pathway [26]; and the RHsCas9 strain, which conditionally expresses Cas9 upon the addition of rapamycin to obtain strains functionally deleted for *mob1* [25].

Human foreskin fibroblasts (HFF) were used as host cells to maintain *T. gondii*. The cell line HFF-1 (SCRC-1041; ATCC, Manassas, VA, USA) was grown in Dulbecco’s Modified Eagle Medium (DMEM) with GlutaMAX Supplement (Gibco; Invitrogen; Thermo Fisher Scientific, Inc., Waltham, MA, USA) supplemented with 10% heat-inactivated fetal bovine serum (FBS; Gibco; Invitrogen; Thermo Fisher Scientific, Inc., Waltham, MA, USA). For passaging, HFF were washed twice with phosphate-buffered saline (PBS; Invitrogen; Thermo Fisher Scientific, Inc., Waltham, MA, USA), detached from the Petri dish using Trypsin-EDTA (0.05%) phenol red (Gibco, Invitrogen; Thermo Fisher Scientific, Inc., Waltham, MA, USA), and resuspended in an appropriate volume of DMEM GlutaMAX 10% FBS. HFF were grown to confluent monolayers to maintain *T. gondii* cultures and were used no further than passage 30. Cell cultures were kept at 37 °C, in a humidified atmosphere with 5% CO_2_. *T. gondii* tachyzoites were cultured using DMEM GlutaMAX, supplemented with 1% FBS.

*T. gondii* tachyzoites were transfected by electroporation using program U-033 of the Nucleofector™ 2b and the Amaxa^®^ Basic Parasite Nucleofector Kit 2 (Lonza, Basel, Switzerland). Approximately 2 × 10^7^ tachyzoites and 10–15 µg of plasmid were used for each transfection. Tachyzoites were selected for successful plasmid integration through drug selection for at least 3 passages with the drug, considering the drug resistance gene present in the plasmid, namely xanthine (40 µg/mL; Sigma, St. Louis, MO, USA) and mycophenolic acid (25 µg/mL; Sigma, St. Louis, MO, USA), pyrimethamine (1 µM; Fluka; Sigma, St. Louis, MO, USA), or chloramphenicol (20 µM; Sigma, St. Louis, MO, USA). Following transfection and drug selection, clonal lines were isolated by limit dilution in 96-well plates seeded with confluent HFF. Wells where a single lysis plaque formed in the host cell monolayer were used to identify clonal lines, which were subsequently characterized.

MOB1-GFP, FLAG-BirA*, FLAG-BirA*-MOB1, and FLAG-MOB1 strains were obtained by the transfection of RH tachyzoites and DNA construct random integration. The MOB1_YFP strain was obtained by the transfection of RHΔku80 tachyzoites and DNA integration through homologous recombination at the *mob1* genomic locus. *Mob1* RHsCas9 strains (*mob1*-wt) were obtained by transfection of RHsCas9 tachyzoites and DNA construct random integration (*pU6-sgRNA1-Dhfr*, *pU6-sgRNA2-Dhfr*, or *pU6-sgRNA3-Dhfr*). *Mob1* functional knockout strains (*mob1*-ko) were obtained by Cas9 activation through the addition of rapamycin (50 nM; Sigma, St. Louis, MO, USA) to the culture medium, followed by clonal line isolation.

### 2.5. TgMOB1 Expression Assay

For the analysis of *mob1* transcript levels during the lytic cycle, freshly egressed 9 × 10^6^ RH tachyzoites were inoculated onto confluent HFF cells in T-25 flasks and kept in standard culture conditions for 2 h. At this point, the wells were washed with PBS to remove extracellular tachyzoites and a fresh culture medium was added to the flasks. Flasks were processed for the recovery of total RNA at determined times after infection to follow the full course of infection: 0 (extracellular, freshly egressed), 4, 24, 38, and 44 (extracellular, freshly egressed) hours. Three independent experiments were performed per assay.

For the comparison of *mob1* transcript levels in wild-type versus TgMOB1-overexpressing strains, freshly egressed 9 × 10^6^ RH, FLAG-MOB1, or FLAG-BirA-MOB1 tachyzoites were inoculated onto confluent HFF cells in T-25 flasks and kept in standard culture conditions for 2 h. At this point, the wells were washed with PBS to remove extracellular tachyzoites and a fresh culture medium was added to the flasks. Flasks were processed for the recovery of total RNA 24 h after inoculation. Three independent experiments were performed per assay.

RNA was extracted using the E.Z.N.A. Total RNA Kit I, following the manufacturer’s instructions. Genomic DNA contamination was eliminated with RNase-free DNase Set I. Complimentary DNA was synthesized using SuperScript III Reverse Transcriptase following the manufacturer’s instructions. The cDNA synthesis was performed using 1 μg of total RNA and random primers (Invitrogen; Thermo Fisher Scientific, Inc., Waltham, MA, USA). This cDNA was processed for qPCR using PerfeCTa SYBR Green SuperMix with ROX reference dye, on an ABI Prism 7700 Sequence system (Applied Biosystems, Waltham, MA, USA), for 40 cycles. Relative quantification was performed using the standard curve method, using *α-tubulin* as the *T. gondii* endogenous control. Standard curve samples were run in triplicate and test samples were run in duplicate. Data are presented as the mean ± standard error (SE) of the three independent experiments. The data were processed for statistical analysis and data visualization using the Microsoft Excel spreadsheet application and were analyzed using an analysis of variance (ANOVA), followed by Tukey multiple comparisons of means for a significance value (α) of 0.05 using R [45]. Specific primers used for cDNA quantification are described in Table S2.

### 2.6. Invasion and Replication Efficiency Assays

To evaluate the effect of MOB1 overexpression on tachyzoite invasion, 5 × 107 freshly egressed parasites were incubated with and without Shd for six hours at 37 °C. The parasites were then inoculated onto confluent HFF seeded on 24-well plates over glass coverslips and kept in standard culture conditions for one hour. To evaluate the effect of *mob1* knockout on tachyzoite replication, 106 freshly egressed parasites were inoculated onto confluent HFF seeded on 24-well plates over glass coverslips and kept in standard culture conditions for one hour. For both assays, following the one-hour invasion period, wells were washed four times with abundant PBS to remove extracellular tachyzoites, a fresh culture medium was added to the wells, and the invaded tachyzoites were allowed to grow for 24 h (counting from inoculation). Following the 24 h period, the wells were washed twice with PBS, fixed with ice-cold methanol (Fluka; Sigma, St. Louis, MO, USA) for 20 min at −20 °C, washed twice with PBS again, and kept in PBS at 4 °C until processed for an immunofluorescence assay.

To evaluate the effect of TgMOB1 overexpression on tachyzoite replication, 105 freshly egressed parasites were incubated with and without Shd for six hours at 37 °C. The parasites were then inoculated onto confluent HFF monolayers cultured on glass coverslips in 24-well plates and kept in standard culture conditions with and without Shd for 24 h. To evaluate the effect of the lack of TgMOB1 expression on tachyzoite replication, 106 freshly egressed parasites were inoculated onto confluent HFF monolayers grown on glass coverslips in 24-well plates and kept in standard culture conditions for one hour. At this point, the wells were washed four times with PBS to remove extracellular tachyzoites, a fresh culture medium was added to the wells, and the invaded tachyzoites were allowed to grow for 24 h (counting from inoculation). For both assays, following the 24 h period, wells were washed twice with PBS, fixed with ice-cold methanol for 20 min at −20 °C, washed twice with PBS again, and kept in PBS at 4 °C until processed for an immunofluorescence assay. Cells were stained with polyglutamylated tubulin monoclonal antibody (to stain the apical complex), polyclonal sera for *T. gondii* surface proteins, and 4′,6-diamidino-2-phenylindole (DAPI; Sigma, St. Louis, MO, USA). The number of parasites inside each parasitophorous vacuole was determined in at least five randomly chosen fields per coverslip using the 20× objective. Each parasitophorous vacuole was assigned to a class following the expected 2n progression (i.e., 2, 4, 8, 16, and 32). One tachyzoite vacuole was not included in the count. Whenever the number of tachyzoites per vacuole did not correspond to the 2n progression, the number of tachyzoites was rounded to the nearest class. The number of vacuoles was counted in at least five randomly chosen fields per coverslip using the 20× objective. Data are presented as the mean number of parasitophorous vacuoles ± SE. In the replication assays, the total number of counted vacuoles was normalized to 100% for each strain. Three independent experiments were performed per assay, including three technical replicates per experiment. The data were processed for statistical analysis and data visualization using the Microsoft Excel spreadsheet application and were analyzed using an analysis of variance (ANOVA), followed by Tukey multiple comparisons of means for a significance value (α) of 0.05 using R [45].

### 2.7. Immunofluorescence Assays

Freshly egressed tachyzoites were inoculated onto confluent HFF monolayers grown on glass coverslips in 24-well plates and were kept under standard culture conditions as indicated. For labeling, the wells were washed twice with PBS, and cells were fixed with ice-cold methanol (Fluka; Sigma, St. Louis, MO, USA) for 20 min at −20 °C, washed twice with PBS again, and kept in PBS at 4 °C until processed. Fluorescence staining was performed at room temperature. Fixed cells were incubated with the blocking solution (PBS 3% (*w*/*v*) bovine serum albumin (BSA; Calbiochem; Sigma, St. Louis, MO, USA)), for 30 min. Primary and secondary antibodies were incubated with the preparations for one hour and diluted in blocking solution. Following primary and secondary antibodies incubation, the fixed cells were washed twice with PBS. Prior to the application of secondary antibodies, the preparations were washed with PBS 0.1% (*v*/*v*) Tween 20 (Merck, Darmstadt, Germany). DNA staining was achieved by assembling the coverslips on glass slides with DAPI Fluoromount-G Mounting Medium (SouthernBiotech, Birmingham, AL, USA). Antibodies used for the immunofluorescence assays are described in Table S3. Slides were imaged through a Leica DMR microscope equipped with a Leica DFC340FX camera with a 20× objective or 100× oil-immersion objective. Alternatively, confocal Z-series stacks were acquired on a Yokogawa CSU-X Spinning Disk confocal, mounted on a Leica DMi8 microscope, with a 100× 1.4NA oil-immersion objective, using the 405 nm, 488 nm, and 561 nm laser lines, an Andor iXon Ultra EMCCD 1024 × 1024 camera, and Metamorph software (Molecular Devices). Images were processed with the ImageJ software [46].

### 2.8. Western Blot

To obtain protein extracts, freshly egressed parasites were centrifuged at 800× *g* for 10 min at room temperature (RT). The pellet was washed twice with PBS. Parasites were lysed in sucrose-based lysis buffer (250 mM sucrose (Sigma, St. Louis, MO, USA), 100 mM NaCl (Merck, Darmstadt, Germany), 50 mM 4-(2-hydroxyethyl)-1-piperazineethanesulfonic acid (HEPES; Sigma, St. Louis, MO, USA) pH 7.6, 2 mM ethylenediaminetetraacetic acid (EDTA; Bio-Rad, Hercules, CA, USA), 0.1% (*v*/*v*) Triton X-100 (Sigma, St. Louis, MO, USA), 1× Halt Protease and Phosphatase Inhibitor Cocktail EDTA-free (Thermo Fisher Scientific, Inc., Waltham, MA, USA)) and centrifuged at 16,000 × *g* for 20 min at 4 °C. Protein was quantified by the Bradford Protein assay and prepared for gel electrophoresis by the addition of 5× protein loading buffer (250 mM Tris-HCl pH 6.8 (Calbiochem; Sigma, St. Louis, MO, USA), 30% (*v*/*v*) glycerol (Invitrogen; Thermo Fisher Scientific, Inc., Waltham, MA, USA), 16% (*v*/*v*) β-mercaptoethanol (Sigma, St. Louis, MO, USA), 4% (*w*/*v*) sodium dodecyl sulfate (SDS; Bio-Rad, Hercules, CA, USA), 0.2% (*w*/*v*) bromophenol blue). The samples were heated at 100 °C for 10 min, then 50 µg of each sample was loaded onto 10–15% polyacrylamide gels for SDS polyacrylamide gel electrophoresis (SDS-PAGE) (25 mAmp per gel). Following protein migration, the proteins were transferred to a nitrocellulose membrane (0.45 µm; GE Healthcare, Chicago, IL, USA) in transfer buffer (192 mM glycine (Merck, Darmstadt, Germany), 25 mM Tris (Calbiochem; Sigma, St. Louis, MO, USA), 10% (*v*/*v*) methanol (VWR, Radnor, PA, USA)), overnight at 30 V, using a wet Mini Trans-Blot transfer system (Bio-Rad, Hercules, CA, USA).

Membranes were incubated with blocking solution (PBS 5% (*w*/*v*) Molico skimmed milk (Nestlé, Vevey, Switzerland)) overnight at 4 °C. After blocking, membranes were probed with a primary antibody (Table S3), diluted in blocking solution, for one hour with orbital shaking at RT, followed by three wash steps with PBS 0.1% (*v*/*v*) Tween 20. Membranes were then probed with a horseradish peroxidase (HRP)-labeled secondary antibody, diluted in blocking solution, for one hour with orbital shaking at RT, followed by three wash steps with PBS. HRP was detected with Amersham ECL Prime Western Blotting Detection Reagent (GE Healthcare, Chicago, IL, USA), according to the manufacturer’s instructions, in the imager ChemiDoc XRS+ (Bio-Rad, Hercules, CA, USA). The antibodies used for Western blot are described in Table S3.

For the detection of biotinylated proteins, an alternative protocol was used. Following SDS-PAGE and protein transfer as described above, membranes were incubated with blocking solution (PBS 2.5% (*w*/*v*) BSA (NZYTech, Lisbon, Portugal), 0.4% (*v*/*v*) Triton X-100) overnight at 4 °C. After blocking, membranes were probed with Streptavidin-HRP (Invitrogen; Thermo Fisher Scientific, Inc., Waltham, MA, USA), diluted in blocking solution (1:50,000), for one hour with orbital shaking at RT, followed by three wash steps with PBS. HRP was detected with Amersham ECL Prime Western Blotting Detection Reagent, according to the manufacturer’s instructions, in the imager ChemiDoc XRS+.

### 2.9. Biotinylation Assays

The BioID method [27] employs proximity-dependent biotin labeling, allowing for the identification of interacting and neighboring proteins in their native cellular environment by proximity labeling, and enabling the identification of the proximity interactome, including stable and dynamic interactions. To achieve this for TgMOB1, freshly egressed 2 × 107 FLAG-BirA* or FLAG-BirA*-MOB1 tachyzoites were inoculated onto HFF confluent cells in T-75 flasks in DMEM GlutaMAX™ 1% FBS. Following 14 h of host cell infection, biotin was added for a final concentration of 150 µM and incubated for 56 h. At this point, the tachyzoites had fully lysed the host cell monolayer, and the culture medium was harvested to recover the parasites. The medium was centrifuged at 800× *g* for ten minutes at RT. The resulting pellets were washed twice with PBS and centrifuged at 800× *g* for 10 min at RT to obtain medium-free parasite pellets. The pellets were lysed in a sucrose-based lysis buffer (described in 2.8) to a ratio of approximately 1:15 (*w*/*v*) and 5% of the volume was reserved for protein electrophoresis. The protein extracts were incubated with agarose-streptavidin beads overnight at 18 °C using an orbital shaker at 45 rpm (GyroTwister; Labnet, Edison, NJ, USA). Following binding, the beads were washed twice with lysis buffer followed by centrifugation at 2000× *g* for 5 min. Next, the beads were washed three times with PBS and centrifuged at 3220× *g* for 5 min, then 10% of the beads’ volume was separated for assay analysis. After the final centrifugation step, the PBS was removed using a needle and syringe before storage at −35 °C. The frozen agarose beads were analyzed through mass-spectrometry by the i3S Proteomics Scientific Platform (Porto, Portugal).

### 2.10. Proteomics

Each sample was reduced and alkylated and processed for proteomics analysis following the solid-phase-enhanced sample-preparation (SP3) protocol as described by Hughes et al. [47]. Enzymatic digestion was performed with Trypsin/LysC (2 µg) overnight at 37 °C at 1000 rpm. Protein identification and quantitation were performed by nanoLC-MS/MS. This equipment is composed of an Ultimate 3000 liquid chromatography system coupled to a Q-Exactive Hybrid Quadrupole-Orbitrap mass spectrometer (Thermo Fisher Scientific, Inc., Waltham, MA, USA). Samples were loaded onto a trapping cartridge (Acclaim PepMap C18 100 Å, 5 mm × 300 µm i.d., 160454; Thermo Fisher Scientific, Inc., Waltham, MA, USA) in a mobile phase of 2% ACN and 0.1% FA at 10 µL/min. After 3 min of loading, the trap column was switched in-line to a 50 cm by 75 μm inner diameter EASY-Spray column (ES803, PepMap RSLC, C18, 2 μm; Thermo Fisher Scientific, Inc., Waltham, MA, USA) at 250 nl/min. Separation was generated by mixing A: 0.1% FA and B: 80% ACN, with the following gradients: 5 min (2.5% B to 10% B), 120 min (10% B to 30% B), 20 min (30% B to 50% B), 5 min (50% B to 99% B) and 10 min (hold 99% B). Subsequently, the column was equilibrated with 2.5% B for 17 min. Data acquisition was controlled by Xcalibur 4.0 and Tune 2.9 software (Thermo Fisher Scientific, Inc., Waltham, MA, USA). The mass spectrometer was operated in data-dependent (dd) positive acquisition mode, alternating between a full scan (*m*/*z* 380–1580) and subsequent HCD MS/MS of the 10 most intense peaks from the full scan (normalized collision energy of 27%). ESI spray voltage was 1.9 kV. Global settings: use lock masses best (*m*/*z* 445.12003), lock mass injection Full MS, chrom. peak width (FWHM) 15 s. Full scan settings: 70 k resolution (*m*/*z* 200), AGC target 3e6, maximum injection time 120 ms. dd settings: minimum AGC target 8e3, intensity threshold 7.3e4, charge exclusion: unassigned, 1, 8, >8, peptide match preferred, exclude isotopes on, dynamic exclusion 45s. MS2 settings: microscans 1, resolution 35 k (*m*/*z* 200), AGC target 2e5, maximum injection time 110 ms, isolation window 2.0 *m*/*z*, isolation offset 0.0 *m*/*z*, spectrum data type profile.

### 2.11. Statistics

The raw data were obtained from three biological replicates for each strain, were processed using Proteome Discoverer 2.4.0.305 software (Thermo Fisher Scientific, Inc., Waltham, MA, USA), and searched against the ToxoDB-46_TgondiiME49 database. The Sequest HT search engine was used to identify tryptic peptides. The ion mass tolerance was 10 ppm for precursor ions and 0.02 Da for fragment ions. The maximum allowed missing cleavage sites was set to 2. Cysteine carbamidomethylation was defined as constant modification. Methionine oxidation and protein N-terminus acetylation were defined as variable modifications. Peptide confidence was set to high. The processing node Percolator was enabled with the following settings: maximum delta Cn 0.05, decoy database search target FDR 1%, validation based on *q*-value. Protein label-free quantitation was performed with the Minora feature detector node at the processing step. Precursor ions quantification was performed at the consensus step with the following parameters: peptides to use unique plus razor, precursor abundance was based on intensity, normalization mode was based on total peptide amount, pairwise protein ratio calculation, hypothesis test was based on t-test (background based). Principal component analysis was performed by comparing the peptides detected in two groups using the Proteome Discoverer software. Two quantification methods were considered: label-free quantification, with data-dependent acquisition, with the protein ratio calculation obtained from a pairwise ratio-based approach; and a Top3 quantification, with the protein ratio calculation obtained from a protein abundance-based approach, in which the calculation of protein abundances was based on the sum of the three most abundant peptides. Peptides were considered with a minimum of six amino acids. A protein was considered detected with a combined false discovery rate experimental *q*-value ≤ 0.05, an abundance ratio adjusted *p*-value ≤ 0.05, and when present in at least two out of three replicates. Proteins that were detected in only one of the biological replicate groups or which were present in statistically significantly higher amounts in one of the biological replicate groups with both quantification methods were considered enriched in either control (FLAG-BirA*) or MOB1 (FLAG-BirA*-MOB1) pull-downs. Proteins that were detected and quantified in all three pull-downs of a biological replicate group and never detected in the other groups were considered high-confidence preys. Proteins that were detected in the MOB1 pull-down with a statistically significant higher fold-change compared to the control pull-down were considered MOB1 enriched.

Functional classification through gene ontology (GO) was performed with OmicsBox software version 2.0.29. GO terms were obtained from a merging of Blast and InterProScan searches. Enrichment analysis was performed to identify over-represented GO terms using Fisher’s exact test (*p* < 0.05). Revigo was used to summarize and visualize GO terms [48]. GO-term frequency was compared to the whole Uniprot database.

## 3. Results

### 3.1. The Toxoplasma gondii Genome Encodes a Single MOB1 Protein Homolog

**Figure 1:**
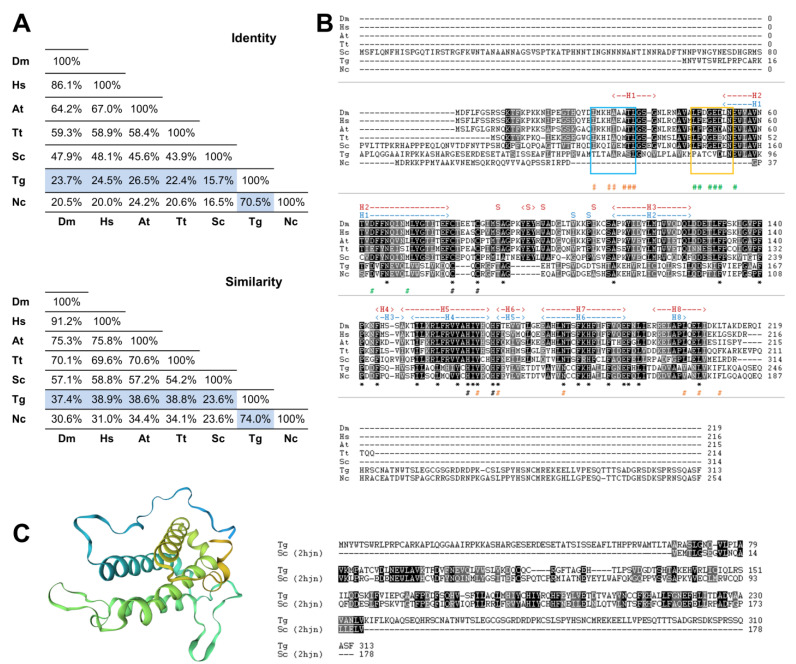
The *Toxoplasma gondii* MOB1 protein is highly conserved. The *T. gondii* MOB1 protein sequence was compared to other eukaryote MOB1 proteins. (**A**) Identity and similarity analysis of MOB1 proteins. The percentages of the *T. gondii* MOB1 versus other eukaryote MOB1 proteins assessed are highlighted in blue. (**B**) Alignment of MOB1 proteins. At the bottom of the alignment, asterisks (*) signal invariant residues. The conserved zinc-binding cysteine (*T. gondii* C105 and C107) and histidine residues (*T. gondii* H188 and H193) are indicated by a black # at the bottom. *S. cerevisiae* MOB1 residues that participate in the NDR–MOB1 interaction are indicated by a green # at the bottom [33]. The region of the NDR kinase binding motif LPXGED is highlighted by a yellow box [33]. ScMOB1 residues that participate in the MOB1 homodimer interaction are indicated by an orange # at the bottom [32]. The region of the ScMOB1 homodimer interaction-conserved residues integrating the helix H1 are highlighted by a light blue box [32]. The *T. gondii* MOB1 secondary structure, predicted by the SWISS-MODEL, is indicated at the top of the alignment in dark blue. The ScMOB1 secondary structure is indicated at the top of the alignment in dark red [32]. (**C**) *T. gondii* MOB1 theoretical 3D model based on the ScMOB179-314 structure (PDB h2jn) and corresponding sequence alignment. A rainbow color scheme from blue (N-terminus) to yellow (C-terminus) is presented. Dm, *Drosophila melanogaster*; Hs, *Homo sapiens* A; At, *Arabidopsis thaliana* A; Tt, *Tetrahymena thermophila*; Sc, *Saccharomyces cerevisiae*; Tg, *Toxoplasma gondii*; Nc, *Neospora caninum*.

To search for the presence of MOB genes in the parasite *T. gondii*, we performed a bioinformatics analysis by searching the ToxoDB.org database using as queries the deduced amino acid sequences of the best-characterized MOB1 proteins, the *H. sapiens* HsMOB1A and the *S. cerevisiae* ScMOB1. This search revealed the presence of one gene coding for a MOB1 protein, TgMOB1 (TGME49_304730). TgMOB1 is predicted to have 313 amino acids and a molecular mass of 34.9 kDa. This protein presents 24.5%/15.7% of identity and 38.9%/23.6% of similarity with the HsMOB1A and ScMOB1 sequences, respectively (Figure 1A). Additionally, we extended our search to other Hippo/MEN/SIN core members to assess the putative conservation of these pathways in *T. gondii* (Figure S1). We identified two NDR kinases that are probable homologs to Dbf/2/20p/LATS1/2 and NDR1/2 kinases, and an STE kinase that could perform a similar function to that of Cdc15p/MST1/2 kinases [49].

Next, we compared the putative MOB1 sequence from *T. gondii* with MOB1 proteins from another eukaryote previously characterized. This comparison revealed that, as expected, TgMOB1 has the highest similarity with the MOB1 sequence from the related apicomplexan parasite *Neospora caninum*, and both diverge substantially in the N-terminal and C-terminal regions from the MOB1 sequences of the other eukaryotes assessed. Still, the central region of TgMOB1 contains the conserved residues corresponding to the MOB kinase activator family domain (*T. gondii* amino acids 69–229, PF03637) (Figure 1B). The zinc-binding cysteine and histidine pairs are conserved in all the species assessed. However, while the *T. gondii* and *N. caninum* MOB1 proteins present a CQC motif, the rest of the assessed species present a CXXXXC motif (Figure 1B). The ScMOB1 homodimer, detected in crystallography studies, is mediated by conserved residues that allow the association of a shallow hydrophobic depression and the amphipathic helix H1 (referred to as H0 in Mrkobrada et al. [32]). These residues are moderately conserved in *T. gondii* (Figure 1B). Of eight ScMOB1 residues identified as participating in the NDR–MOB1 interaction, the four residues located more in the C-terminal are conserved in *T. gondii* (D88, E91, D99, and L106) [33]. Of the more N-terminal residues, the NDR kinase binding motif LPXGED [33] is minimally conserved in *T. gondii* that presents a PATCVD motif instead (Figure 1B). To further analyze TgMOB1, we obtained a *T. gondii* MOB1 theoretical 3D structure model based on the known structure of the ScMOB179-314 region (PDB h2jn) (Figure 1C). The raw alignment included the residues 67–235 (54% coverage), 31% identity, a global model quality estimate (GMQE) of 0.33, and a qualitative model energy analysis distance constraint (QMEANDisCo) global score of 0.55 ± 0.07. The difference in identity score to that reported in Figure 1A occurs because the model alignment uses the ScMOB179-314, lacking the 78 amino acid N-terminal region that is specific to *S. cerevisiae*. The resulting model presents a core domain with an α helix-based structure organized in a bundle, common to MOB proteins and similar to the structure of the template ScMOB179-314. The results of the 3D model indicate high confidence in homology between proteins.

**Figure 2:**
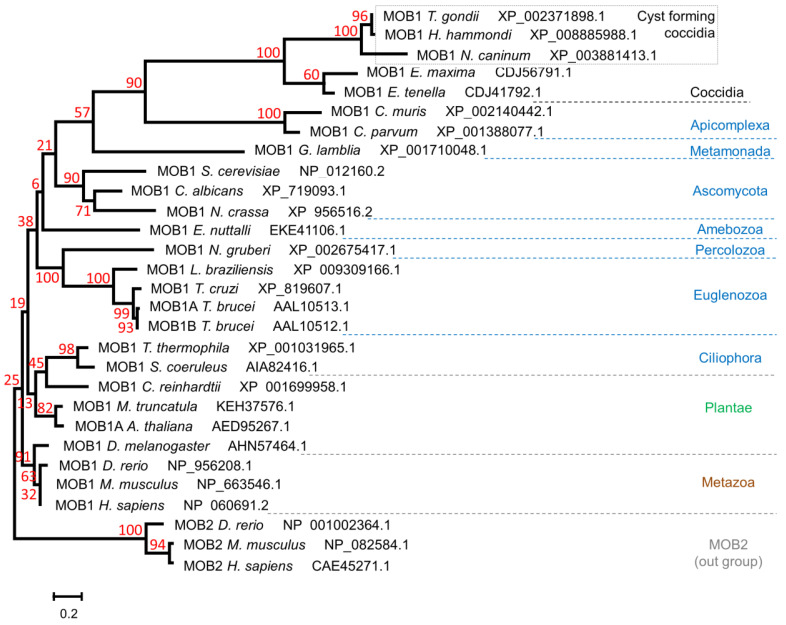
Phylogenetic analysis of MOB1 proteins from different parasitic organisms. The predicted amino acid sequence of *T. gondii* MOB1 and those of different parasitic organisms were compared with MOB1 proteins from model organisms throughout the eukaryotic tree of life. The sequences were aligned and used to perform a maximum likelihood phylogenetic analysis. Kingdom (brown and green), phylum (blue), and ad hoc cluster names (black) are indicated on the right side. Bootstrap values are presented in red. The scale bar indicates the estimated number of amino acid substitutions per site.

In order to investigate the evolutionary relationship between MOB1 proteins from different organisms, we performed a phylogenetic analysis, including parasites as well as model organisms (Figure 2). The first node separates animals from two other groups: (i) the group containing plants and unicellular organisms such as microalgae (*Chlamydomonas reinhardtii*) and ciliates (*T. thermophila*, *Stentor coeruleus*); and (ii) the group containing the remaining organisms. Focusing on the unicellular organisms included in the phylogenetic analysis, we observe that free-living and parasitic organisms are in different clades of the tree. Regarding parasitic organisms, a distinction between the *Trypanosoma* and *Leishmania* species, which belong to the supergroup Excavata, and parasites belonging to the phylum Apicomplexa (*T. gondii*, *Hammondia hammondi*, *N. caninum*, *Eimeria tenella*, *Eimeria maxima*, *Cryptosporidium parvum*, and *Cryptosporidium muris*) was detected. However, the MOB1 sequence from *Giardia lamblia* (Excavata) appears closer to the Apicomplexa than to the Trypanosomatida. The Apicomplexa form a monophyletic group in which cyst-forming coccidia (*T. gondii*, *H. hammondi*, *N. caninum*) appear very closely related and separated from the monoxenous genus *Eimeria*, while *C. muris* and *C. parvum* form a more distant group, mirroring the evolutionary history of these species. To date, no MOB proteins have been identified in hematozoan genomes, namely in *Plasmodium* spp., *Babesia* spp., or *Theileria* spp. [49].

### 3.2. RNA Expression and Gain-of-Function Analyses Suggest That TgMOB1 Is Maintained at Low Levels in the Intracellular Tachyzoite

**Figure 3:**
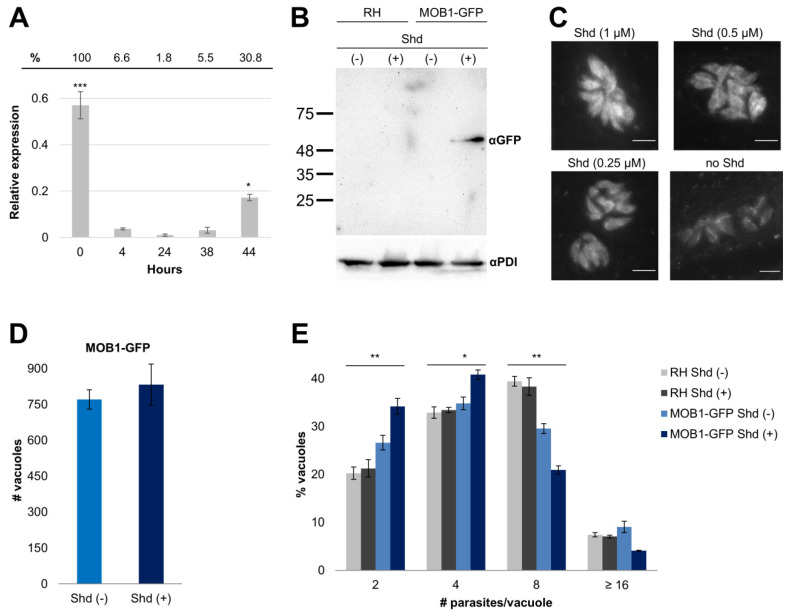
*Mob1* overexpression tachyzoites are viable, presenting a slight delay in replication. (**A**) Freshly egressed RH tachyzoites (wild type for *mob1* expression) present higher levels of *mob1* which dramatically lower after invasion and during active replication inside host cells, after which *mob1* transcript levels increase again in egressed tachyzoites. The percentages at the top represent relative expression levels normalized to a time of 0 h. The *T. gondii* endogenous control used was *α-tubulin*. (**B**) Western blot analysis of the MOB1-GFP strain (free tachyzoites) responding to the addition of Shield1 (Shd), using an anti-GFP antibody (αGFP). The *T. gondii* protein disulfide isomerase (PDI) was used as a loading control (using an anti-PDI antibody, αPDI). Strains are indicated at the top. Numbers on the left represent molecular mass. (**C**) Immunofluorescence analysis of the MOB1-GFP strain with anti-GFP showing lower levels of protein expression in response to decreasing Shd concentrations. (**D**) Invasion assay of MOB1-GFP tachyzoites in the absence and presence of Shd. No difference in the invasion rate was observed (*p* = 0.169). (**E**) Replication assay of RH and MOB1-GFP tachyzoites in the absence and presence of Shd. In MOB1-GFP overexpression background, the replication rate is delayed in the presence of Shd. MOB1-GFP tachyzoites Shd (+) presented significantly more vacuoles, with 2 and 4 parasites, compared to MOB1-GFP tachyzoites Shd (−), RH Shd (−), and RH Shd (+) (differences in means from 6.00 to 14.00, adjusted *p*-values < 0.05), and significantly fewer vacuoles compared to MOB1-GFP tachyzoites Shd (−), RH Shd (−), and RH Shd (+) (differences in means from −17.39 to −8.78, adjusted *p*-values < 0.001). No difference was detected in non-transformed RH tachyzoites Shd (−) and Shd (+) (differences in means from −1.11 to 0.98, adjusted *p*-values = 1). Assays described in (**B**,**D**,**E**) were performed with 1 µM of Shd. Expression, invasion, and replication data are presented as mean ± SE of three independent experiments. *—*p* < 0.05; **—*p* < 0.01; ***—*p* < 0.001.

We characterized the *mob1* transcript levels during RH tachyzoite in vitro infection of HFF cells at 0, 4, 24, 38, and 44 h of infection through a qPCR analysis (Figure 3A). *Mob1* transcript levels are significantly different at increasing time points during infection (F = 78.2, *p* < 0.0001). Free, invasion-ready tachyzoites present higher levels of *mob1* transcripts in comparison with those of tachyzoites inside of host cells (0 h, transcript level considered 100%). In fact, at different times after host-cell invasion, corresponding to tachyzoite replication inside the host cell, we observed a downregulation of the transcript levels (4 h, 6.6%; 24 h, 1.8%; 38 h, 5.5%). The levels of *mob1* transcripts were upregulated in freshly egressed tachyzoites (44 h, 30.8%). To investigate the role of TgMOB1 in *T. gondii*, we assessed the effects of its overexpression in tachyzoites. For this, we constructed a transgenic line expressing the fusion protein TgMOB1-GFP, controlled by the protein destabilization domain ddFKBP. We confirmed that, in the absence of the Shd ligand, no recombinant protein can be detected, while TgMOB1-GFP protein accumulates in the presence of Shd (1 µM), visible as a ~60 kDa band in a Western blot analysis (Figure 3B). Immunofluorescence microscopy shows TgMOB1-GFP accumulating in the cytoplasm, excluded from the nucleus (Figure 3C). This strategy permits us to precisely control the levels of the recombinant protein using different Shd concentrations. We evaluated the invasion and replication efficiencies of TgMOB1-GFP tachyzoites in the absence and in the presence of Shd. We detected no significant variation in invasion efficiency in TgMOB1-GFP-overexpressing tachyzoites (F = 4.92, *p* = 0.169) (Figure 3D). However, we found that TgMOB1-GFP tachyzoites overexpressing MOB1 (with Shd) present a significant delay in replication compared to tachyzoites not overexpressing TgMOB1 (without Shd), while no delay was observed in the untransformed strain, RH, when exposed to Shd in the same conditions (F = 31.64, *p* < 0.0001) (Figure 3E). Similar results regarding replication efficiency were obtained with two additional TgMOB1-GFP clonal lines, highlighting that this phenotype is a specific effect of the TgMOB1-GFP recombinant protein and not the consequence of the random genomic integration of the vector (Figure S2). Overall, the data show a decrease in the replication rate of *T. gondii* tachyzoites upon TgMOB1-GFP overexpression, which is coherent with the observed decreased expression of the *mob1* gene after invasion (Figure 3A). These results support the idea that low levels of *mob1* transcripts are advantageous for the replication of *T. gondii* tachyzoites. However, the effects of TgMOB1 overexpression are mild and do not compromise strain survival.

### 3.3. TgMOB1 Loss-of-Function Tachyzoites Do Not Present Compromised Mitotic Exit or Cytokinesis

To go further in the characterization of TgMOB1, we obtained a *mob1* gene knockout (*mob1*-ko) employing a CRISPR/Cas9 system and using three gRNAs, targeting each of the first three exons of *mob1* (Table S2). We were able to isolate viable clonal lines expressing the three sgRNAs, a stark contrast to other unicellular [3,5,9,50] and multicellular eukaryotes [6,7,51] in which *mob1* was shown to be an essential gene and MOB1 depletion led to cytokinesis defects.

**Figure 4:**
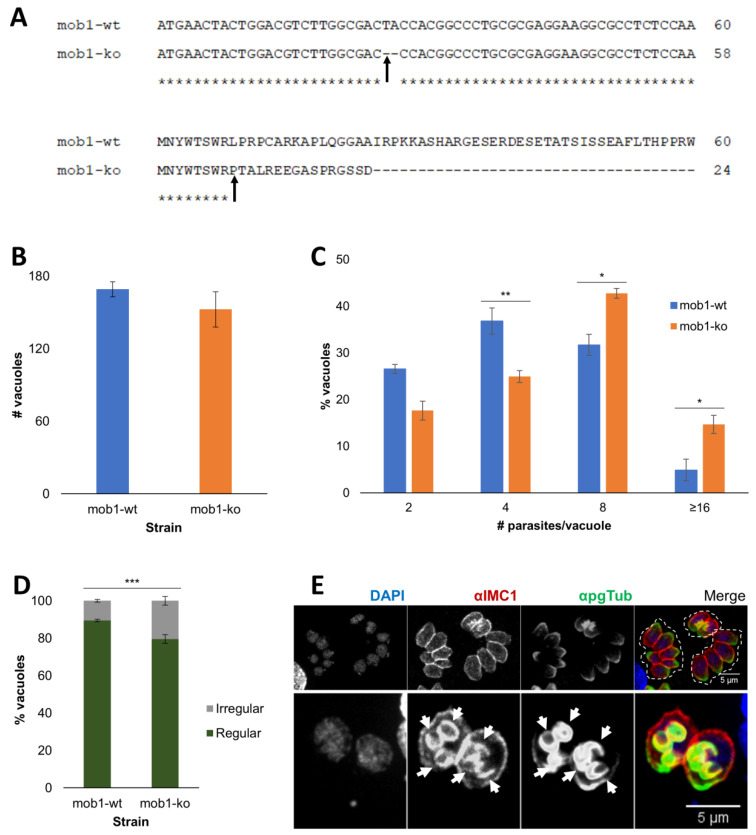
*Mob1* knockout causes a slight increase in tachyzoite replication rates and irregular parasitophorous vacuole occurrence. (**A**) Comparison of sgRNA1 *mob1*-wt and *mob1*-ko strain DNA and predicted protein sequence. The *mob1*-ko predicted protein sequence is truncated by a premature STOP codon. Asterisks signal conserved regions and the arrows indicate the place of sequence disruption. At the bottom of the alignment, asterisks (*) signal invariant residues. (**B**) Invasion assays did not detect significant differences between *mob1*-wt and *mob1*-ko tachyzoites (*p* = 0.3241). (**C**) Replication assays detected significant differences between *mob1*-wt and *mob1*-ko tachyzoites (*p* < 0.0001). *Mob1*-ko tachyzoites present a higher replication rate compared to *mob1*-wt tachyzoites. *—*p* < 0.05; **—*p* < 0.01. (**D**) PV regularity assays detected significantly higher irregular vacuoles in *mob1*-ko tachyzoites compared to *mob1*-wt tachyzoites (*p* = 0.004). ***—*p* < 0.001. (**E**) Immunofluorescence of *mob1*-ko tachyzoites showing regular (two and four tachyzoites) and irregular (six tachyzoites) vacuoles (**top panel**). No defects were observed regarding cytoskeleton morphology. We observed vacuoles with three daughter cells forming inside each mother cell (**bottom panel**, arrows). The inner membrane complex is stained with anti-IMC1 (αIMC1), the tubulin cytoskeleton is stained with anti-polyglutamylated Tubulin (αpgTub), and the nuclei are stained with DAPI. Data are presented as mean ± SE of three independent experiments. *mob1*-wt, RHsCas9 expressing the *mob1* sgRNA1; *mob1*-ko, *mob1* functional knockout expressing the *mob1* sgRNA1; Irregular, vacuoles with numbers of tachyzoites different from 4, 8, 16, or 32; Regular, vacuoles with numbers of tachyzoites equal to 4, 8, 16, or 32.

The TgMOB1 coding sequence was targeted at its beginning to increase the chances of achieving a functional knockout using the CRISPR/Cas9 system, as this promotes disruption at the beginning of the coding sequence, ensuring a low chance of having a truncated functional protein. By sequencing, we identified insertions and deletions (indels) that occurred at the site targeted by the gRNA used, namely three nucleotides before the PAM (*mob1* nucleotide 27 with gRNA1, nucleotide 175 with gRNA2, and nucleotide 208 with gRNA3). To study the effects of *mob1* knockout, we selected the *mob1*-ko clonal lines harboring a maximum protein disruption for each gRNA. From the sgRNA1 *mob1*-ko clonal lines, we selected a clonal line that suffered a ‘TA’ deletion, and from the sgRNA2 and sgRNA3 *mob1*-ko clonal lines, we selected clonal lines that suffered ‘A’ and ‘T’ insertions, respectively (Figure 4A and Figure S2). Following the confirmation that our clonal lines had mutations expected to lead to an inactive TgMOB1 protein, we proceeded with the characterization of the respective phenotypes comparing the parental clonal line, RHsCas9 tachyzoites expressing a *mob1* sgRNA without Cas9 activation (*mob1*-wt), to the respective knockout clonal line, RHsCas9 tachyzoites expressing the same *mob1* sgRNA isolated after Cas9 activation (*mob1*-ko). Regarding tachyzoite invasion efficiency, the *mob1*-ko strain expressing the sgRNA1 did not present significant differences in comparison to that of the respective *mob1*-wt strain (F = 1.682, *p* = 0.3241) (Figure 4B), while *mob1*-ko tachyzoites presented an increase in replication rate compared to that of *mob1*-wt tachyzoites (F = 19.77, *p* < 0.0001) (Figure 4C). We also estimated the percentage of irregular vacuoles, i.e., the vacuoles that contain a number of parasites per vacuole different from 2n, which indicates abnormal tachyzoites replication. In *mob1*-ko tachyzoites the number of irregular vacuoles (20.47%) was significantly higher than that found in *mob1*-wt tachyzoites (10.54%) (F = 33.97, *p* = 0.0004) (Figure 4D). The analysis of the *mob1*-ko clonal lines expressing the sgRNA2 and sgRNA3 revealed a similar increase in the replication rate and in the PV number regularity phenotypes as detected for sgRNA1 expressing tachyzoites, which strongly indicate that the phenotypes detected are specific to TgMOB1 loss-of-function and are not the result of an off-target effect of a single sgRNA (Figure S3). No morphological defects were observed in dividing or non-dividing *mob1*-ko tachyzoites when staining the microtubule cytoskeleton (Figure 4E). However, we observed dividing *mob1*-ko tachyzoites forming three daughter cells instead of the standard two daughter cells per mother, suggesting an impact on tachyzoite cell division mechanisms during asexual replication.

### 3.4. Novel Potential MOB1-Interacting Proteins Identified through the TgMOB1 BioID System

**Figure 5:**
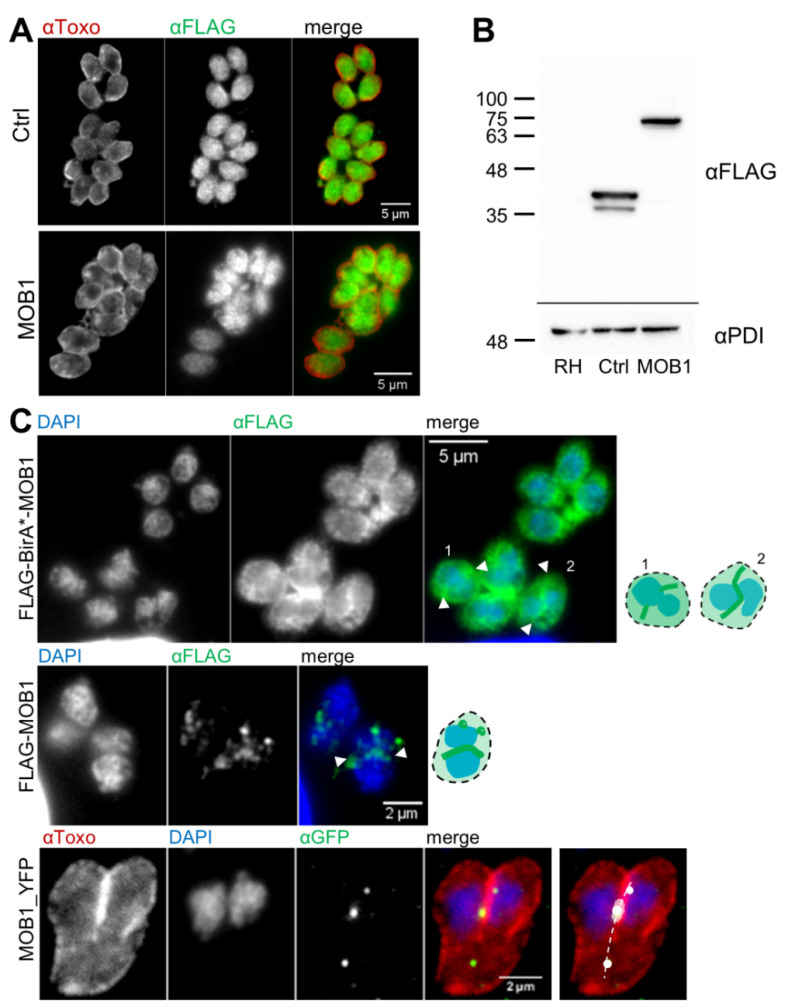
MOB1 accumulates between the two newly formed nuclei during endodyogeny. (**A**) The localizations of the predicted recombinant proteins of the control (Ctrl) and MOB1 clonal lines were detected through immunofluorescence, showing a similar distribution within the cell. *T. gondii* surface proteins were identified with polyclonal sera (αToxo) while the FLAG-tagged recombinant proteins were identified using anti-FLAG (αFLAG). (**B**) The expressions of the recombinant proteins by the Ctrl and MOB1 clonal lines were confirmed by Western blot analysis, presenting molecular masses compatible with the predicted 41 kDa for FLAG-BirA* and 76 kDa for FLAG-BirA*-MOB1, respectively. The protein disulfide isomerase (PDI) was used as a loading control. Numbers on the left represent molecular mass in kDa. (**C**) Subcellular localization of recombinant and endogenous MOB1 proteins at the end of mitosis. FLAG-BirA*-MOB1 accumulates between the two newly divided nuclei at the end of mitosis when the new nuclei finish segregation but are still very close, forming a distinct linear pattern (arrowheads). During the rest of the cell cycle, FLAG-BirA*-MOB1 was observed in the cytoplasm excluded from the nucleus. The recombinant protein FLAG-MOB1 showed a pattern similar to that observed for FLAG-BirA*-MOB1, accumulating between the two daughter nuclei (arrowheads). During the rest of the cell cycle, FLAG-MOB1 was also observed in the cytoplasm. Endogenous MOB1 was detected in a punctate linear pattern between the two daughters, in a pattern similar to that observed with the recombinant MOB1 proteins. A graphical representation of the observed MOB1 accumulation is represented in the detail on the right for each panel. The recombinant proteins FLAG-BirA*-MOB1 (driven by a strong promoter) and FLAG-MOB1 (driven by a moderate promoter) were analyzed using anti-FLAG (αFLAG). The subcellular localization of endogenous MOB1 was observed using the MOB1_YFP strain and staining with anti-GFP (αGFP) and polyclonal anti-*T. gondii* surface proteins (αToxo). DNA is stained with DAPI. Ctrl, FLAG-BirA*; MOB1, FLAG-BirA*-MOB1.

In order to shed light on the potential interaction partners of TgMOB1 and its putative functional role in *T. gondii* tachyzoites, we employed the proximity-dependent biotin-labeling method BioID [27], a tool used to identify the proximity interactome of several *T. gondii* proteins [52,53], in *T. gondii* tachyzoites overexpressing TgMOB1. As a first step, immunofluorescence microscopy was used to determine the localization of recombinant TgMOB1 in *T. gondii* strains with high and moderate TgMOB1 overexpression. The recombinant proteins expressed by the BioID control (FLAG-BirA*) and MOB1 (FLAG-BirA*-MOB1) clonal lines present a similar intracellular distribution with a cytoplasmic pattern (Figure 5A). Western blot analysis confirmed the expression of the recombinant proteins with the predicted molecular masses (FLAG-BirA* ~41 kDa, FLAG-BirA*-MOB1 ~76 kDa) (Figure 5B). The recombinant FLAG-BirA*-MOB1 protein (driven by a strong promoter) presented a cytoplasmic subcellular localization excluded from the nucleus during most of the cell cycle. However, in dividing tachyzoites, the protein accumulated with a curved linear pattern at the region of the axis that separates the two newly formed nuclei, in a transitory and cell cycle-regulated manner (Figure 5C, top panel). This time-dependent localization was mostly detected after nuclei segregation, when nuclei were still very small and in close proximity, while cell division was still ongoing (Figure 5C top panel, arrowheads). The same patterns of subcellular localization were observed for the FLAG-MOB1 recombinant protein driven by a moderate promoter (Figure 5C, middle panel). Additionally, the qPCR analysis shows markedly distinct *mob1* transcript levels when comparing the control (endogenous *mob1*) and MOB1-overexpression strains (Figure S4). The recombinant protein FLAG-MOB1 was detected at the expected molecular mass of ~35 kDa through Western blot (Figure S4). In order to confirm this subcellular localization and validate our recombinant proteins, we constructed an endogenously tagged version of TgMOB1 by obtaining a modified Δku80 strain harboring a YFP tag at the C-terminal end of the *mob1* gene, MOB1_YFP. This strain revealed a discrete accumulation of a punctate signal between the two newly divided nuclei, forming a linear pattern (Figure 5C, bottom panel). This accumulation was aligned with the axis of cell division, being visible only at this precise moment of the cell cycle, consistent with the end of mitosis. These data show that the mid-daughter nuclei accumulation of recombinant TgMOB1 proteins largely mirrored the subcellular localization of the endogenous TgMOB1. Thus, these strains expressing recombinant TgMOB1 proteins were suitable for studies on TgMOB1 interaction partners.

**Figure 6:**
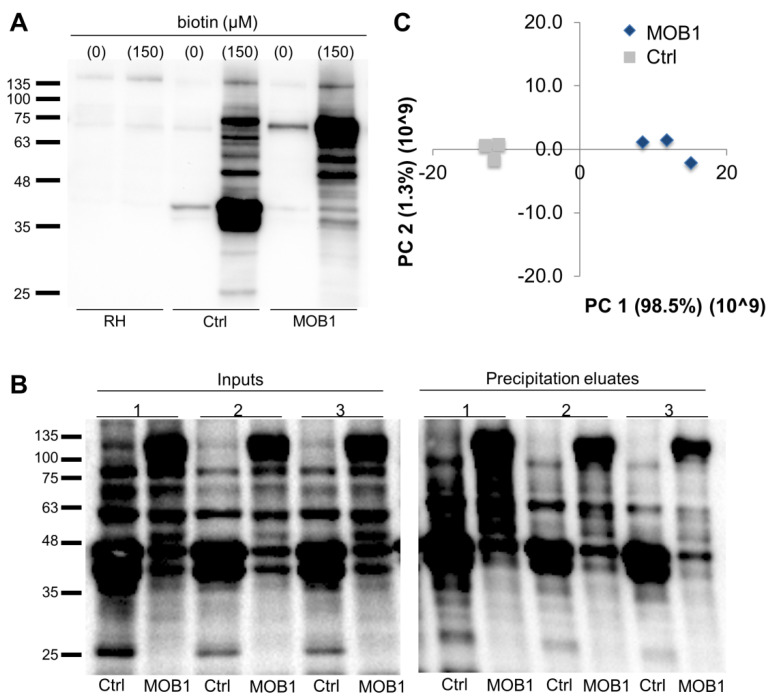
Evaluation of the MOB1 BioID system. (**A**) Western blot using streptavidin-HRP confirmed the in vivo protein biotinylation by the BirA*-recombinant proteins. Ctrl and MOB1 protein extracts present substantially higher amounts of biotinylated proteins when biotin is added to the culture medium, compared to wild-type RH tachyzoites and to when no biotin is supplied. Numbers on the left represent molecular mass in kDa. (**B**) Evaluation of the precipitation of the BioID assay using streptavidin-HRP. The precipitation successfully isolated biotinylated proteins from control and MOB1 protein extracts of three biological replicates. Numbers on the left represent molecular mass in kDa. (**C**) A principal component analysis of the detected peptides shows a clear clustering of Ctrl and MOB1 pull-downs according to their biological replicate group. The first factor (PC 1) accounts for 98.5% of the variance between samples. Ctrl, FLAG-BirA*; MOB1, FLAG-BirA*-MOB1.

Using streptavidin-HRP, we observed that the MOB1 BioID system was able to generate a pattern of biotinylated proteins in vivo. The protein extracts obtained from the control and MOB1 clonal lines when biotin was added to the culture medium present high levels of biotinylated proteins, in comparison to those observed when protein extracts were obtained in the absence of biotin or to that of the parental RH strain (Figure 6A). The biotinylation patterns of the three biological replicates used for the large-scale purification and identification of the MOB1 proximity interactome are shown in Figure 6B. A principal component analysis of the detected peptides shows that the control and MOB1 pull-downs cluster together according to their biological group and independently from the other group, with the principal component 1 accounting for 98.5% of the variance between samples (Figure 6C). We detected a total of 3274 proteins, of which 97 were uniquely or significantly more detected in the MOB1 pull-down and corresponding to 494 unique peptides (Table 1, File S1).

Among the 3274 proteins, we detected the recombinant proteins FLAG-BirA* and FLAG-BirA*-MOB1. Most notably, we also detected the endogenous MOB1 with one unique peptide with high confidence, corresponding to the most N-terminal endogenous MOB1 peptide cleaved at the first arginine residue, indicating that the FLAG-BirA*-MOB1 recombinant protein co-localized with the MOB1 endogenous protein in the tachyzoite cell (Figure S5). These proteins share several peptides, which confounds quantification, therefore abundance values were not considered for MOB1, FLAG-BirA*, or FLAG-BirA*-MOB1. Among the full MOB1 proximity interactome, 34 proteins (~35%) are annotated as uncharacterized, in line with the ~30% estimated as uncharacterized for the full *T. gondii* proteome. While MOB proteins are considered kinase activator proteins, only four proteins harboring kinase domains were detected in the TgMOB1 proximity interactome. The 20 most abundant proteins among the MOB1 pull-down high-confidence prey group—proteins quantified in the three MOB1 pull-downs and not detected in the control pull-downs—are listed in Table 2. Notably, the TGME49_309890 uncharacterized protein is the most abundant protein among this high-confidence group, considering both the label-free and the Top3 quantification methods. TGME49_309890 is a 333.8 kDa protein with an alpha-beta-hydrolase domain at its N-terminal end (Figure S5). The next most abundant high-confidence preys are an uncharacterized protein with no clear domains (TGME49_206540) and an SWI2/SNF2-containing protein, RAD26 protein (TGME49_263140). The top 20 MOB1 high-confidence group also included an alpha-beta-hydrolase domain-containing protein (TGME49_223510) and an aspartyl aminopeptidase (TGME49_297970). An additional DNA repair protein, RAD51 (TGME49_272900), was also detected in the TgMOB1 interactome. The proteins among the MOB1-enriched group that present an abundance fold-change above 5 are listed in Table 3. The three proteins with the highest fold-changes among MOB1-enriched proteins are an uncharacterized protein (TGME49_234560) with an N-terminal domain related to the low conductance mechanosensitive channel YnaI, an anaphase-promoting complex subunit1 (APC1, TGME49_275410), and an endonuclease/exonuclease/phosphatase family protein (TGME49_243590).

A gene ontology enrichment analysis did not detect GO terms significantly over-represented in the TgMOB1 proximity interactome in comparison to the *T. gondii* ME49 genome (Figure S4). The most representative GO terms include the cellular component terms integral component of membrane (GO:0016021) and proteasome complex (GO:0000502), and the biological process terms cell cycle (GO:0007049), microtubule-based process (GO:0007017), and vesicle-mediated transport (GO:0016192). The proteasome complex and cell cycle GO terms both relate to the anaphase-promoting complex subunit1 (APC1), characterized in other organisms as part of the anaphase-promoting complex/cyclosome, an E3 ubiquitin ligase complex that promotes mitosis progression through ubiquitin-dependent protein degradation [54]. Notably, a ubiquitin family protein (TGME49_254600) was also detected as part of the high-confidence preys while the cell-cycle-associated protein kinase DYRK was detected among the MOB1-enriched proteins.

## 4. Discussion

The function of MOB1 in cell division appears to be deeply conserved throughout the eukaryotic tree of life [4,5,7,8,12,13]. Among the proteins that constitute the Hippo signaling pathway, MOB1 is the one that appears earlier in eukaryotes, supporting a core role for this protein [18].

Interestingly, we identified only one MOB1 protein in *T. gondii*. Most eukaryotes present more than one MOB [5,19]. To the best of our knowledge, the only organism with only one MOB1 protein studied so far was the ciliate *Tetrahymena thermophila*, but the *T. thermophila* genome also encodes a MOB4 protein [55], while no additional MOB family protein was identified in *T. gondii*. Amino acid sequence and phylogenetic analyses identify TgMOB1 as a partially conserved protein, a feature that appears to be common among apicomplexan parasites. A search for orthologues of the most well-characterized MOB1 partners, such as LATS and MST proteins, suggested that genes coding for potentially related proteins are present in the *T. gondii* genome [49]. However, these proteins were not identified in the TgMOB1 proximity biotinylation interactome. Indeed, among the proteins identified as TgMOB1 high-confidence preys, the most abundant was an uncharacterized protein harboring an alpha-beta-hydrolase domain. Among the TgMOB1 proximity interactome, we identified five proteins with hydrolase/peptidase domain-containing proteins, and three were classified in the top 20 most abundant of the high-confidence MOB1-specific group, suggesting that, in *Toxoplasma*, TgMOB1 may present a higher affinity for alpha-beta-hydrolase domain-containing proteins. This putative distinct affinity for binding partners may be related to the differences in conserved residues detected in the TgMOB1 sequence. The loss-of-function studies also indicate a divergent role for TgMOB1, as the absence of this protein does not hinder the mitotic exit or cytokinesis of tachyzoites in vitro, a feature highly conserved in other unicellular and multicellular eukaryotes studied to date, in which the knockout of *mob1* genes led to cell division defects [4,8,11,56]. However, our results are consistent with the results of a previously conducted genome-wide loss-of-function screen using CRISPR/Cas9 relative to *mob1*, which indicated that *mob1* was not an essential gene in the lytic cycle of *T. gondii* RH tachyzoites (phenotype data available at ToxoDB.org [57]).

Nevertheless, our data also point to some conserved features of TgMOB1. The TgMOB1 proximity interactome included proteins related to the ubiquitin-proteasome complex and cell cycle progression. Notably, the regulation of MOB proteins by the ubiquitin-proteasome complex has been reported in mammals, namely through the ubiquitination of MOB1 and MOB2 proteins [58,59,60,61,62,63]. DYRK family proteins and APC1, also identified through proximity biotinylation, have been shown to regulate the Hippo pathway, where MOB1 proteins play an integral role [64,65,66]. Furthermore, in *H. sapiens*, the DNA repair protein HsRAD50 interacts with HsMOB2 [67,68], while we detected two RAD proteins in the TgMOB1 interactome. Interestingly, the TgMOB1 proximity interactome, gain-of-function phenotype, and loss-of-function phenotype are more coherent with the functions reported for MOB1 proteins of metazoans in the context of Hippo tumor-suppression signaling, which leads to cell overgrowth and the occurrence of tumors when mutated/depleted [19,69]. Moreover, during embryonic development, when tissues and organs are actively growing, MOB1 and the rest of the Hippo pathway core proteins are repressed and only activated when organs reach their proper size [70]. This pathway is regulated by extracellular signals, namely mechanical and chemical stimuli, perceived through the plasma membrane [71]. This specific role of the Hippo pathway in cell proliferation control is an essential issue for multicellular organisms that present a supracellular organization structured in tissues and organs. Despite how *T. gondii* is a unicellular organism, this parasite only divides inside a parasitophorous vacuole that is established in the host cell cytoplasm, where parasites are in close association and must cooperate. This is particularly necessary for the tissue cyst, a modified parasitophorous vacuole produced by the slowly replicating bradyzoite stage of cyst-forming coccidia that remains viable in the host’s tissues and enables recrudescence and transmission through carnivorism. Thus, the parasite replication (or eventual regulation of cell division *versus* cell death) has to be able to adjust to spatial constraints, positioning the *T. gondii* mode of proliferation between those of free-living unicellular and tissue-organizing cells. Our data suggest that, although the MOB1 function may not be fully conserved in *T. gondii*, mechanisms similar to those that regulate MOB proteins in other organisms, namely in metazoans, may also be present in *T. gondii*.

MOB1 proteins frequently localize at organelles/structures essential for cell cycle progression and associated with the cell division plane [49], often being necessary for appropriate cell morphogenesis and polarity maintenance [4,8,50,56,72]. In tachyzoites, the subcellular localization of TgMOB1 strongly resembles the localization described for Myosin B/C during tachyzoite cell division. The myosin B/C gene, involved in daughter cell budding and abscission, codes for two proteins that result from alternative splicing, which accumulate in what the authors describe as distinct structures or lamellae extending from the periphery between the daughter cells [73]. Notably, *T. gondii* Myosin B/C was identified as a TgMOB1 high-confidence prey in the current study (File S1). In *Medicago sativa* root tip cells, MsMOB1 is present in grains and fibrillary structures in the cytoplasm during the cell cycle, being more prominent in the G2 and mitosis phases and accumulating at the mid-plane between the daughter cells from late anaphase to cytokinesis, marking septum formation during cytokinesis [74]. However, the functional significance of this localization is not yet fully understood.

The TgMOB1 subcellular localization and the phenotypes of the gain-of-function and loss-of-function suggest that this protein is involved in *T. gondii*’s replication, although not presenting the canonical MOB1 role in mitotic exit. Tachyzoites undergo asexual expansion through endodyogeny, where the nuclear and budding cycles occur in parallel to ensure proper daughter cell formation [75]. *T. gondii* accomplishes this through conserved cell cycle checkpoints that allow for the co-progression of these cycles when conditions are met. TgMOB1 could be involved in the regulation of tachyzoite replication synchronicity, with increased TgMOB1 levels leading to replication delay and a lack of this protein, producing an increase in asynchronous replication. Alternatively, the increase in replication and in irregular vacuoles detected in *mob1*-ko tachyzoites could be due to a shift from the traditional tachyzoite cell-division mechanism, endodyogeny (one mother cell produces two identical daughter cells) to multiple daughter cell formation, an event we documented in this clonal line. The asexual expansion phase of the *T. gondii* development in the cat intestine occurs in the form of endopolygeny, and this is considered a rare phenomenon in tachyzoite proliferation; still, multiple daughter cell formation has been described in RH and P-strain in vitro cultured tachyzoites with varying frequencies (0.5–10%) [76].

Our data indicate that TgMOB1 is present in the tachyzoite replicative stage at very low levels. Interestingly, RNAseq datasets available at ToxoDB.org show that *mob1* transcripts are expressed at lower levels in the asexual stages (tachyzoites and bradyzoites) and at higher levels in sexual stages (feline enterocytes and sporonts) [23,77], suggesting that TgMOB1 may have different roles depending on the parasite life-cycle stage. However, applicable in vitro models to study *T. gondii*’s sexual development are still lacking, hampering research in this phase of the life cycle. Di Genova et al. [78] recently published an exciting study showing the paramount role of linoleic acid in *T. gondii* sexual reproduction and definitive host specificity, suggesting a functional in vitro model to research *T. gondii*’s sexual development which could be used in the near future. The TgMOB1 gain-of-function and loss-of-function strains developed for this study are useful tools for investigations on the role of TgMOB1 in other stages of the life cycle of this apicomplexan.

## 5. Conclusions

In conclusion, we present evidence that the involvement of MOB proteins in regulatory mechanisms previously described in other eukaryotes may be only partially conserved in the apicomplexan parasite *T. gondii*. In contrast to members of the MOB1 family characterized in unicellular and multicellular organisms to date, the TgMOB1 expressed in *T. gondii* tachyzoites exhibits highly unusual features, especially with regard to its role in mitotic exit and cytokinesis, but also with respect to its putative interaction partners identified through BioID and proteomics analysis. Furthermore, the TgMOB1 gain-of-function and loss-of-function phenotypes, caused by effects on the *T. gondii* replication process, are more in line with respective features in multicellular eukaryotes, with this gene being differentially regulated throughout the *T. gondii* life cycle. Future studies should focus on the role of TgMOB1 in the context of *T. gondii*’s sexual development, which will likely become more feasible with the development of model systems that allow for performing this work in reproducible in vitro conditions.

## Figures and Tables

**Table 1:** Overall statistics of proteome dataset.

	MOB1	Control	Total Proteins
Unique peptides (#)	494	608	27,163
Identified proteins (#)			
FDR confidence high (in MOB1 pulldown only)	84 (39)	80	3128
FDR confidence medium	12	18	141
FDR confidence low	1	0	5
total	97	98	3274

The proteomes were analyzed as described in Materials and Methods. MOB1, pull-down performed with biotinylated FLAG-BirA*-MOB1 tachyzoite protein extracts; Control, pull-down performed with biotinylated FLAG-BirA* tachyzoite protein extracts; #, number; FDR, false discovery rate.

**Table 2:** Twenty most abundant proteins identified with high FDR confidence, only in the MOB1-pull down, in all three analyses.

Accession	Annotation	Abundance (×10^5^)	
		Average	SD
TGME49_309890	Uncharacterized protein	102.45	18.13
TGME49_206540	Uncharacterized protein	15.77	0.63
TGME49_263140	SWI2/SNF2-containing protein RAD26	11.17	2.65
TGME49_254600	Ubiquitin family protein	8.27	1.15
TGME49_225120	Uncharacterized protein	7.34	0.45
TGME49_265010	Glutamate 5-kinase domain-containing protein	7.33	2.20
TGME49_224110	Adhesion regulating molecule region protein, putative	5.53	0.15
TGME49_312410	Uncharacterized protein	4.27	0.36
TGME49_215420	SNARE protein	3.40	1.27
TGME49_216920	Mediator complex subunit MED8	3.07	0.48
TGME49_260450	DEAD/DEAH box helicase domain-containing protein	3.03	0.57
TGME49_223510	AB hydrolase-1 domain-containing protein	3.01	0.50
TGME49_271600	Uncharacterized protein	2.90	0.88
TGME49_272670	Peptidase family M3 protein	2.82	0.95
TGME49_272230	Heat shock protein hslv, putative	2.78	1.76
TGME49_203710	AP2 domain transcription factor AP2VIIa-4	2.59	1.15
TGME49_297970	Aspartyl aminopeptidase	2.55	0.75
TGME49_215250	Thiamine diphosphokinase	2.49	0.17
TGME49_309050	Uncharacterized protein	2.45	0.15
TGME49_262780	FHA domain-containing protein	2.18	0.35

Abundances calculated through the Top3 method. SD, standard deviation.

**Table 3:** Proteins quantified in MOB1 and control pull-downs with fold change above 5.

Accession	Annotation	Fold Change
TGME49_234560	Uncharacterized protein	25.00
TGME49_275410	Anaphase-promoting complex subunit1	16.67
TGME49_243590	Endonuclease/exonuclease/phosphatase family protein	13.70
TGME49_229500	Uncharacterized protein	13.33
TGME49_204280	Cell-cycle-associated protein kinase DYRK, putative	12.50
TGME49_268320	Uncharacterized protein	10.64
TGME49_313600	DDHD domain-containing protein	9.43
TGME49_222090	Uncharacterized protein	9.17
TGME49_264030	Aminotransferase, putative	8.77
TGME49_210778	Hemimethylated DNA binding domain-containing protein	8.62
TGME49_281400	Phosphofructokinase domain-containing protein	8.26
TGME49_210345	Uncharacterized protein	7.75
TGME49_265390	Uncharacterized protein	7.63
TGME49_246990	Uncharacterized protein	6.99
TGME49_204160	Eukaryotic translation initiation factor 2A	6.62
TGME49_254210	RNA recognition motif-containing protein	6.41
TGME49_213415	Uncharacterized protein	6.37
TGME49_305610	Uncharacterized protein	6.33
TGME49_310790	Uncharacterized protein	5.71
TGME49_228630	Uncharacterized protein	5.35

## Data Availability

The mass spectrometry proteomics data have been deposited to the ProteomeXchange Consortium via the PRIDE [79] partner repository with the dataset identifier PXD028781.

## Supplementary Materials

The following are available online at https://www.mdpi.com/article/10.3390/biology10121233/s1, Figure S1: Analysis of homologs of MEN/SIN/Hippo pathway members in Toxoplasma gondii. Figure S2: Evaluation of MOB1-GFP overexpression in tachyzoite replication efficiency; Figure S3: Evaluation of in vitro phenotypes of m*ob1* knockout tachyzoites expressing sgRNA2 and sgRNA3; Figure S4: Western blot and qPCR analysis of wild type and m*ob1* overexpression strains; Figure S5: Proximity biotinylation interactome analysis. Table S1: Accession numbers of the MOB proteins used in the phylogenetic analysis; Table S2: Sequences of gRNAs and primers used in this study; Table S3: Antibodies used for this work; File S1: Proteomics data.
