# Supplementary material for: Characterization of a MOB1 Homolog in the Apicomplexan Parasite Toxoplasma gondii
Source: Biology (Basel). 2021 Nov 26;10(12):1233. doi: 10.3390/biology10121233 (PMC8698288; doi:10.3390/biology10121233)
Supplement: Supplementary file 1 [file biology-10-01233-s001.zip › biology-1463695-supplementary/Figures S1-S5.pdf]

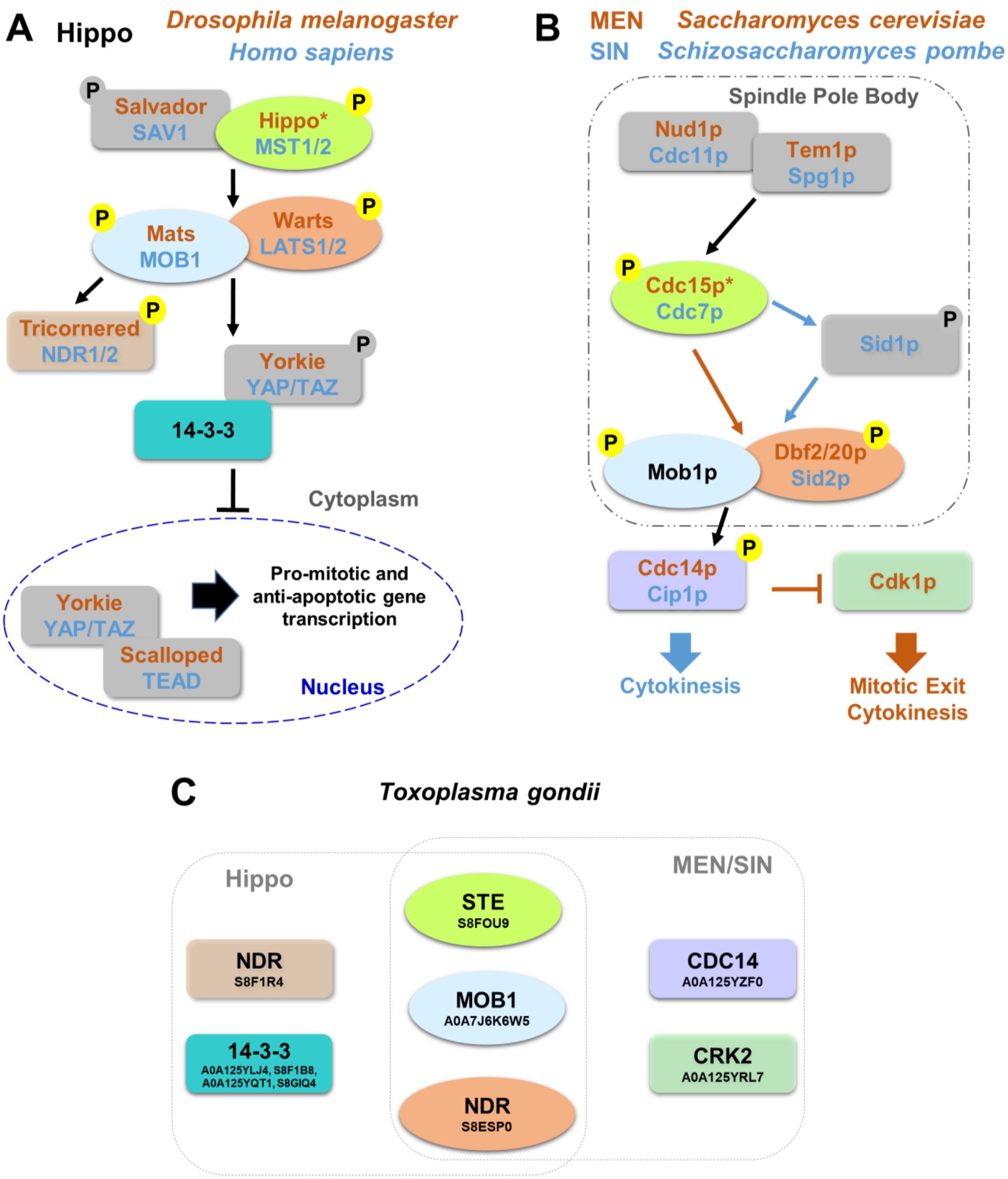


**Figure S1. Analysis of homologs of MEN/SIN/Hippo pathway members in *Toxoplasma gondii*.**

Homolog proteins are represented with oval nodes of the same color (yellow, Salvador/SAV1/Tem1p/Spg1p; green, Hippo/MST1/2/Cdc15p/Cdc7p; blue, Mats/MOB1/Mob1p; orange, Warts/LATS1/2/Dbf2p). (A) Representation of the Hippo pathway in *Drosophila melanogaster* (orange) and in *Homo sapiens* (blue). The upstream Salvador/SAV1 protein that activates the first kinase Hippo/MST1/2 was not identified in *T. gondii*. We identified possible *T. gondii* homologs for Hippo/MST1/2, Warts/LATS1/2, and Mats/MOB1, the Hippo core kinase module. The *T. gondii* genome also codes for four 14-3-3 type proteins that act by sequestering the Yorkie/YAP/TAZ effector proteins in the cytoplasm, inhibiting Hippo activation. *T. gondii* homologs for Yorkie/YAP/TAZ were not identified. (B) Representation

of the Mitotic Exit Network (MEN) in *Saccharomyces cerevisiae* (orange) and Septation Initiation Network (SIN) in *Schizosaccharomyces pombe* (blue). The upstream Nud1p/Cdc11p and Tem1p/Spg1p were not identified in T. gondii while the members of core kinase module Cdc15p/Cdc7p, Dbf2/20p/Sid2p, and Mob1p, common to the Hippo pathway, were identified. The MEN/SIN effector proteins Cdc14p/Cip1p and Cdk1p are also present in *T. gondii*. C) Visualization of the *T. gondii* proteins identified as possible homologs of MEN/SIN/Hippo pathway members. Uniprot identifiers are provided under the protein designation. The 14-3-3, CDC14 and CRK2 proteins typically participate in several essencial signaling pathways and have been previously studied in *T. gondii*. *There are some controversies in the literature regarding whether to consider Cdc15 and Hippo as homologs. This scheme is in accordance with Hergovich 2017 [16]. Figure adapted with permission under the Creative Commons Attribution License from Delgado et al. 2020 [49].

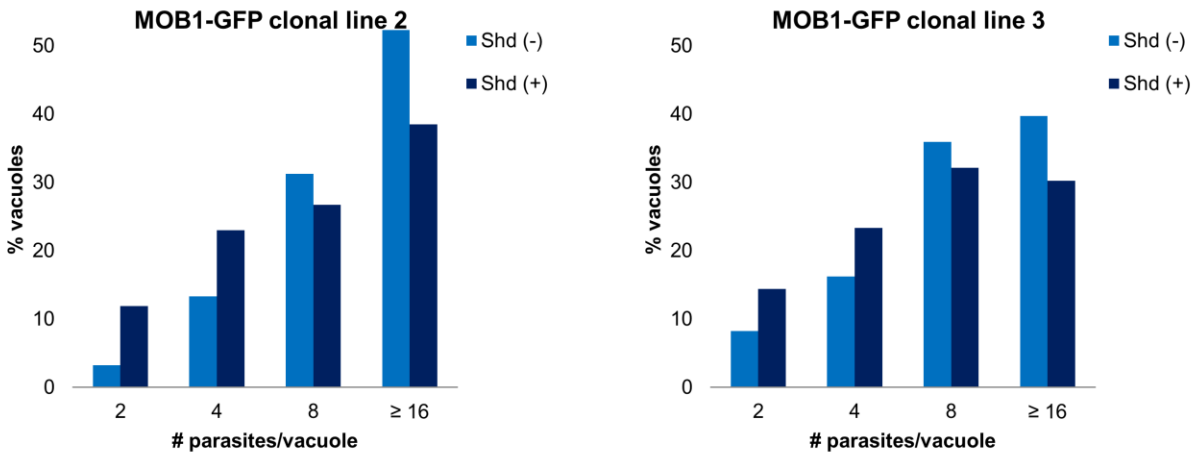


**Figure S2. Evaluation of MOB1-GFP overexpression in tachyzoite replication efficiency**

Replication assay of two additional MOB1-GFP clonal lines in the absence and presence of Shd. MOB1-GFP tachyzoites present a slower replication rate in the presence of Shd, an effect that is consistently observed in different clonal lines obtained through random integration of the *p5RT70-ddFkbp-myc-mob1-gfp-hx* vector. The data represent one independent experiment. HFF cells were inoculated with $5x10^6$ tachyzoites.

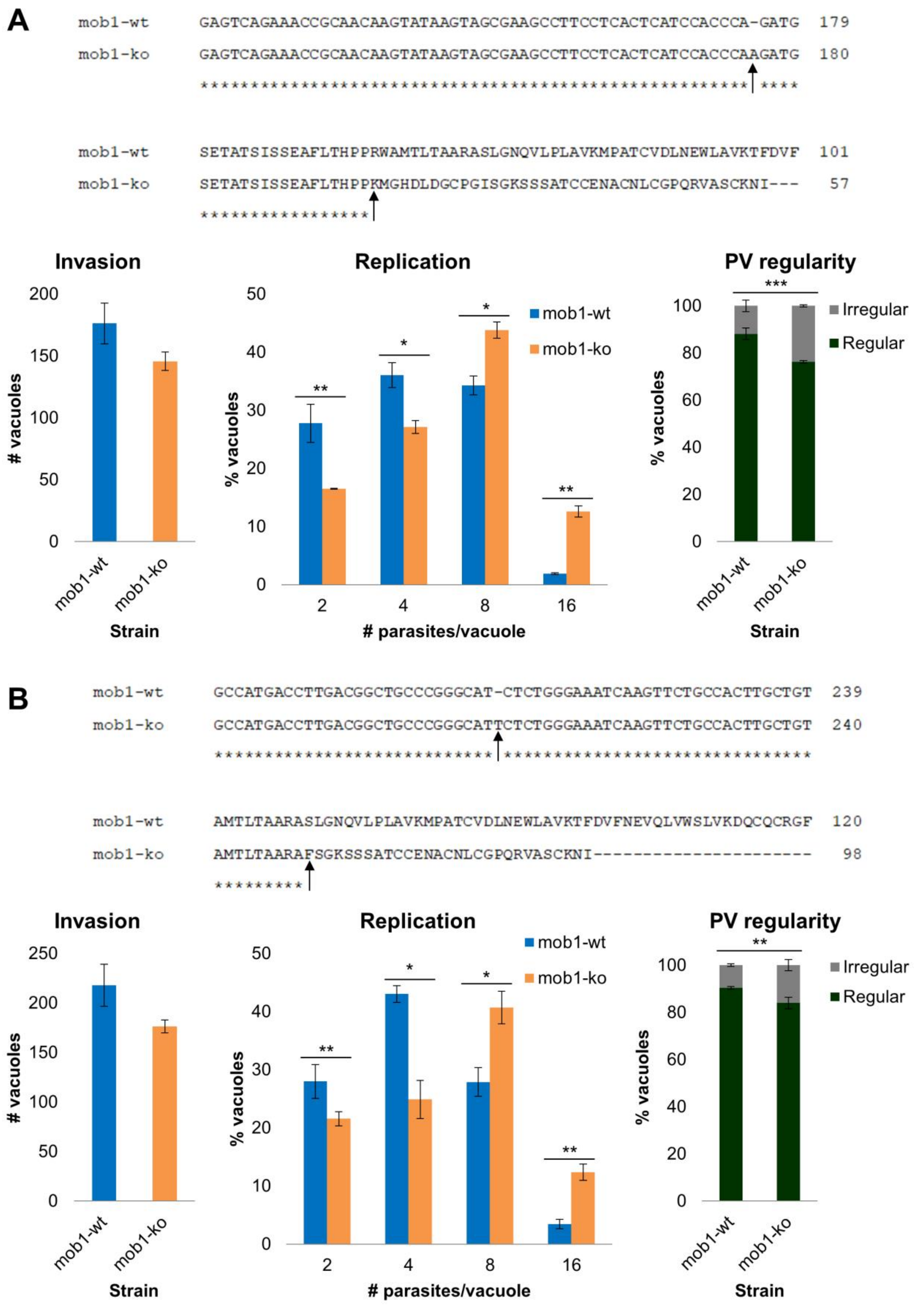


**Figure S3. Evaluation of *in vitro* phenotypes of m*ob1* knockout tachyzoites expressing sgRNA2 and sgRNA3**

*Mob1*-ko tachyzoites expressing the *Mob1* sgRNA2 (A) or sgRNA3 (B) were analyzed for its invasion efficiency, replication efficiency, and PV regularity in comparison to the respective control *mob1*-wt tachyzoites. **A)** Comparison

of sgRNA2 *mob1*-wt and *Mob1*-ko strain DNA and predicted protein sequence. The *Mob1*-ko predicted protein sequence is truncated by a premature STOP codon. Asterisks signal conserved regions and the arrows indicate the place of sequence disruption. Invasion assays did not detect significant differences between *mob1*-wt and *Mob1*-ko tachyzoites (p= 0.1661). Replication assays detected significant differences between *mob1*-wt and *Mob1*-ko tachyzoites (p<0.0001) with *Mob1*-ko tachyzoites presenting a higher replication rate compared to *mob1*-wt tachyzoites. PV regularity assays detected significantly higher irregular vacuoles in *Mob1*-ko tachyzoites compared to *mob1*-wt tachyzoites (p=0.0001). **B)** Comparison of sgRNA3 *mob1*-wt and *Mob1*-ko strain DNA and predicted protein sequence. The *Mob1*-ko predicted protein sequence is truncated by a premature STOP codon. Asterisks signal conserved regions and the arrows indicate the place of sequence disruption. Invasion assays did not detect significant differences between *mob1*-wt and *Mob1*-ko tachyzoites (p= 0.1350). Replication assays detected significant differences between *mob1*-wt and *Mob1*-ko tachyzoites (p<0.0001) with *Mob1*-ko tachyzoites presenting a higher replication rate compared to *mob1*-wt tachyzoites. PV regularity assays detected significantly higher irregular vacuoles in *Mob1*-ko tachyzoites compared to *mob1*-wt tachyzoites (p=0.0062). Data are presented as mean ± SE of three independent experiments. *mob1*-wt, RHsCas9 expressing the *Mob1* sgRNA2 or sgRNA3. *Mob1*-ko, *Mob1* functional knockout expressing the *Mob1* sgRNA2 or sgRNA3. Irregular, vacuoles with a number of tachyzoites different from 4, 8, 16, or 32. Regular, vacuoles with a number of tachyzoites equal to 4, 8, 16, or 32.

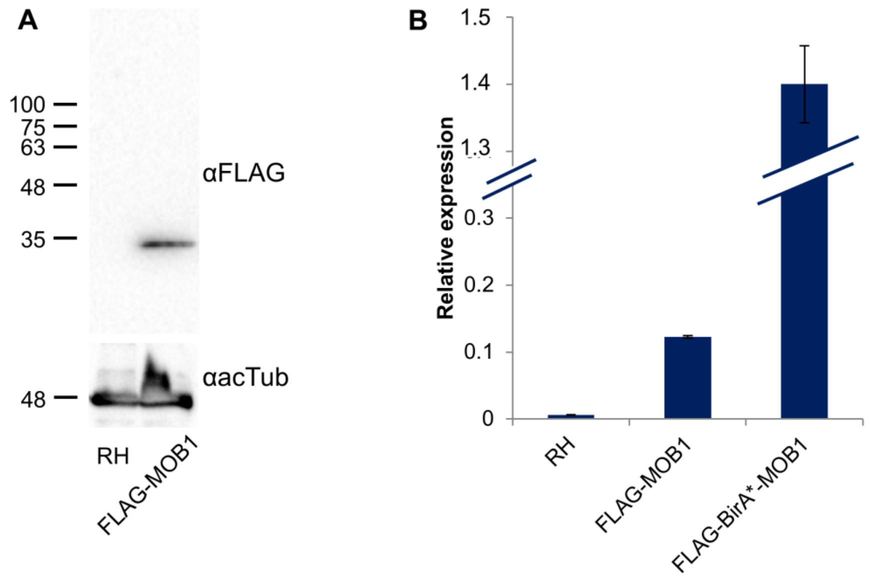


**Figure S4. Western blot and qPCR analysis of wild type and *mob1* overexpression strains.**

A) Western blot analysis of protein extracts of FLAG-MOB1 tachyzoites using anti-FLAG (αFLAG) antibody. Acetylated tubulin (αacTub) was used as loading control. **B)** Analysis of *Mob1* expression in wild type (RH) and *mob1* recombinant strains FLAG-MOB1 (moderate *morn1* promoter) and FLAG-BirA*-MOB1 (strong *p5RT770* promoter). The levels of *mob1* expression of MOB1 recombinant strains are substantially higher then in the RH strain and correlate to the MOB1 protein expression levels detected through Western blot.

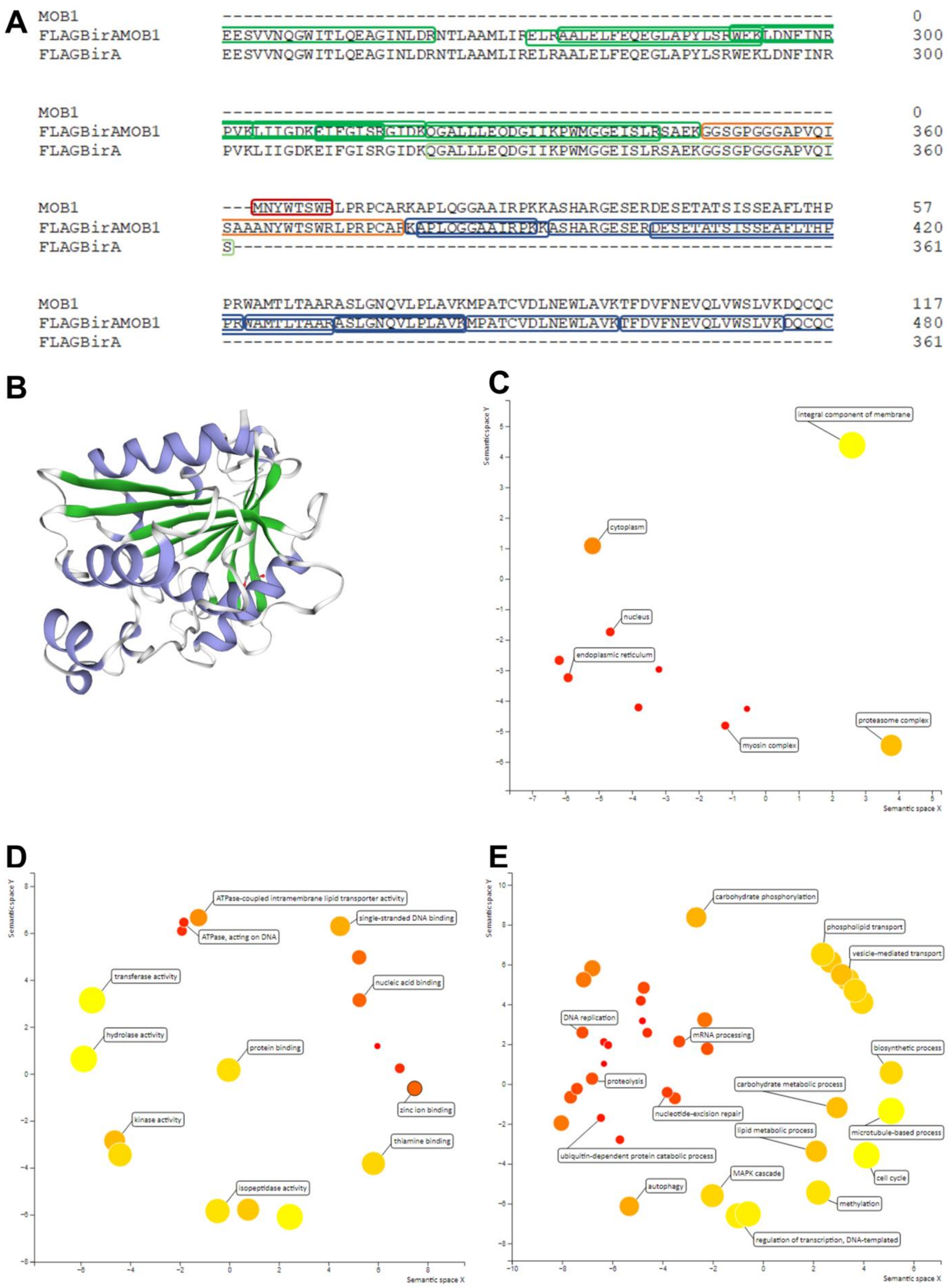


**Figure S5. Proximity biotinylation interactome analysis.**

**A)** Peptides identified belonging to the MOB1, FLAG-BirA*-MOB1, and FLAG-BirA* proteins. Peptides are identified by colored boxes. The peptide unique to the endogenous MOB1 protein is shown in red. Peptides common to FLAG-BirA*-MOB1 and FLAG-BirA* are shown in dark green while peptides common to MOB1 and FLAG-BirA*-MOB1 are shown in dark blue. Peptides unique to FLAG-BirA* are shown in light green while peptides unique to FLAG-BirA*-MOB1 are shown in orange. Common peptides (between MOB1 and FLAG-BirA*-MOB1 or FLAG-BirA*-MOB1 and FLAG-BirA*) are shown imposed only on FLAG-BirA*-MOB1. When one peptide is fully contained by another, only the larger peptide is shown. **B)** Theoretical 3D model of the N-terminal alpha-beta-hydrolase domain of TGME49_309890. Homology modelling for TGME49_309890 identified the proline iminopeptidase 1x2e.1.A as the template most closely related to this domain, with a raw alignment including the residues 224-475 (7% coverage), 17% identity, and a QMEANDisCo global score of 0.43±0.05. The resulting model presents a structure based on alternating α helices and beta sheets. The α helices are represented in blue, beta strands are represented in green, and loops are represented in white. **C)** Cellular component GO terms identified in the MOB1 interactome. **D)** Molecular function GO terms identified in the MOB1 interactome. **E)** Biological process GO terms identified in the MOB1 interactome. GO terms in C, D, and E were summarized using Revigo with color and circle size indicating representativeness of the GO terms within the dataset relative to the whole Uniprot database (yellow and larger circle represents more unique GO terms, red and smaller circles represent less unique GO terms).
