# Supplementary material for: Characterization of a MOB1 Homolog in the Apicomplexan Parasite Toxoplasma gondii
Source: Biology (Basel). 2021 Nov 26;10(12):1233. doi: 10.3390/biology10121233 (PMC8698288; doi:10.3390/biology10121233)
Supplement: Supplementary file 1 [file biology-10-01233-s001.zip › biology-1463695-supplementary/Table S1.pdf]

Table S1. Accession numbers of the MOB proteins used in the phylogenetic analysis.

Species	Accession number	Uniprot identifier
	**MOB1 proteins**	
Toxoplasma gondii	XP_002371898.1	A0A125YPY8
Hammondia hammondi	XP_008885988.1	
Neospora caninum	XP_003881413.1	F0VB04
Eimeria maxima	CDJ56791.1	U6LYH3
Eimeria tenella	CDJ41792.1	U6KUG1
Cryptosporidium muris	XP_002140442.1	B6AD89
Cryptosporidium parvum	XP_001388077.1	A3FQM2
Giardia lamblia	XP_001710048.1	A8B2T9
Saccharomyces cerevisiae	NP_012160.2	P40484
Candida albicans	XP_719093.1	Q5AC22
Neurospora crassa	XP_956516.2	
Entamoeba nuttalli	EKE41106.1	K2GES9
Naegleria gruberi	XP_002675417.1	D2VKE6
Leishmania braziliensis	XP_009309166.1	
Trypanosoma brucei	AAL10513.1	Q95UM8
Trypanosoma brucei	AAL10512.1	Q95UM9
Trypanosoma cruzi	XP_819607.1	Q4DZ03
Tetrahymena thermophila	XP_001031965.1	I7LT46
Stentor coeruleus	AIA82416.1	A0A060BK44
Chlamydomonas reinhardtii	XP_001699958.1	A8I3T3
Medicago truncatula	KEH37576.1	B7FND8
Arabidopsis thaliana	AED95267.1	Q9FHI1
Drosophila melanogaster	AHN57464.1	A0A0B4KGK3
Danio rerio	NP_956208.1	Q7ZV70
Mus musculus	NP_663546.1	Q921Y0
Homo sapiens	NP_060691.2	Q9H8S9
	**MOB2 proteins**	
Danio rerio	NP_001002364.1	Q6DHC8
Mus musculus	NP_082584.1	Q8VI63
Homo sapiens	CAE45271.1	Q70IA6
