# Supplementary material for: Characterization of a MOB1 Homolog in the Apicomplexan Parasite Toxoplasma gondii
Source: Biology (Basel). 2021 Nov 26;10(12):1233. doi: 10.3390/biology10121233 (PMC8698288; doi:10.3390/biology10121233)
Supplement: Supplementary file 1 [file biology-10-01233-s001.zip › biology-1463695-supplementary/Table S2.pdf]

Table S2. Sequences of gRNAs and primers used in this study

**Sequences of gRNAs used to obtain *Mob1* functional knockouts**		
gRNA ID	Target sequence (5' - 3')	Annealing location
gRNA1	GGACGTCTTGGCGACTACCA	exon 1
gRNA2[1]	GCCTCACTCATCCACCCAGAT	exon 2
gRNA3	GACGGCTGCCCGGGCATCTC	exon 3
**Sequences of primers used for RNA characterization and expression analysis**		
Primer ID	Sequence (5' - 3')	Purpose
TgGapdh1_qPCR_F	CGTGGAGGTTTTGGCGATC	Quantification, amplification
TgGapdh1_qPCR_R	GACTTCGCCGGGGTAGTG	Quantification, amplification
TgATub_qPCR_F	CGCCTGCTGGGAGCTCTT	Quantification, amplification
TgATub_qPCR_R	GAAGGTGTTGAAGGCGTCG	Quantification, amplification
TgMob1_qPCR_F	CTGCCACATCTACAGACAAC	Quantification, amplification
TgMob1_qPCR_R	GAGTGGTACGGAGACAATG	Quantification, amplification
seqRNA_MOB1_F	CGGAGTTTCCTTTCCACG	Isolation, amplification, sequencing, quantification
seqRNA_MOB1_R	TACGCATCTACAGAGAAACT	Isolation, amplification, sequencing, quantification
**Sequences of primers used to develop *Mob1* overexpresion tachyzoites**		
Primer ID	Sequence (5' - 3')	Purpose
Mob1 F BamHI	CGGGATCCATGAACTACTGGACGTCTTGG	GFP-MOB1 cloning
Mob1 R EcoRV	CCCGATATCAAAACTGGCCTGAGAGCTC	GFP-MOB1 cloning
Mob1-GFP F NotI	ATAAGAATGCGGCCGCATGAACTACTGGACGTCTTGG	GFP-MOB1 cloning
Mob1-GFP R BamHI	CGGGATCCTTACTTGTACAGCTCGTCCATG	GFP-MOB1 cloning
Mob1 F int	GAAGAAAGCGTCCCATGCAC	Colony testing, sequencing
Mob1 R int	CACGAGATTTGCCACAGCAG	Colony testing, sequencing
Mob1 F int2	CGAAGTCCAGTTGGTGTGGA	Colony testing, sequencing
Mob1 R int2	GCAGTTCGAGTGGTACGGAG	Colony testing, sequencing
Mob1 3 seq	CTAAGTGTTCATTGTCTCCG	Sequencing
Mob1 5 seq	GTGGATGAGTGAGGAAGGC	Sequencing
FLAGBirAMob1 F AflII	AATCGCCTTAAGATGGACTACAAAGACGATGAC	BioID cloning
FLAGBirAMob1 R NdeI	GGAATTCCATATGTCAAAAACTGGCCTGAGAGC	BioID cloning
FLAGBirA R NdeI	GGAATTCCATATGTCAAGATATCTGTACAGGCGCG	BioID cloning
vector F1	TGTGCTGCAAGGCGATTA	Colony testing, sequencing
vector R1	ACTGGGACTGCGAACAGC	Colony testing, sequencing
vector F2	TAAAGGCGGCGAATATTAGC	Colony testing, sequencing

BirA F int	GACAGCACCAACCAGTACCT	Colony testing, sequencing
BirA R int	AGTTGTCCAGCTTCTCCCAC	Colony testing, sequencing
BirA5’ seq	GTTCACCGTGCCCGGCAA	Sequencing
BirA3’ seq	GGAGCAGGACGGCATCATC	Sequencing
FLAGMob1 F BamHI	GGATCCATGGACTACAAAGACGATGACGATAAAAACTACTGGACGTCTTGGC	morn1FMOB1 cloning
FLAGMob1 R NdeI	GGAATTCCATATGTCAAAAACTGGCCTGAGAGC	morn1FMOB1 cloning
morn1FLAGMob1 F	ACCGTTGTCCACCAGATCATGGACTACAAAGACGATGAC	Cloning (ligation independent)
morn1Mob1 R	CGGGCAGCTTCTGGCGCGTCAAAAACTGGCCTGAGAGC	Cloning (ligation independent)
morn1 F	GTATCTCCTGTCTTGAATTA	Colony testing, sequencing
morn1 R	GCAACTCGCTTTCGTTC	Colony testing, sequencing
Mob1 F BamHI	CGGGATCCATGAACTACTGGACGTCTTGG	GST-MOB1 cloning
Mob1 R XhoI	CGCTCGAGTCAAAAACTGGCCTGAGAGC	GST-MOB1 cloning

Sequences of primers used to develop *Mob1* knockout tachyzoites using splitCas9

Primer ID	Sequence (5' - 3')	Purpose
gRNA1_Mob1_pFext	GGTCTGCTTGTGATGCTGAA	Clonal line testing
gRNA1_Mob1_pRext	CATGACGCTACGAGGATCAA	Clonal line testing
gRNA1_Mob1_pFint	ATTGCTTTCCCGTGATAGTCC	Sequencing, clonal line testing
gRNA1_Mob1_pRint	TTCGACTCCAAGGAACAGAG	Sequencing, clonal line testing
gRNA2_Mob1_pFext	AGGCAATCCGAATCCTCTTT	Clonal line testing
gRNA2_Mob1_pRext	TTCACAGCAAGTGGCAGAAC	Clonal line testing
gRNA2_Mob1_pFint	TGGCGAATCTGTGCCTATAA	Sequencing, clonal line testing
gRNA2_Mob1_pRint	TGGTTCCAGTCAAAGCGATA	Sequencing, clonal line testing
gRNA3_Mob1_pFext	CATTTGTCGTTGCTTGCTGT	Clonal line testing
gRNA3_Mob1_pRext	ACGTCAAATGCTGAGGGAAG	Clonal line testing
gRNA3_Mob1_pFint	ACCTCCCAACTTCCCAGAGT	Sequencing, clonal line testing
gRNA3_Mob1_pRint	TTAAAGTGTCATCGTGCAGTCA	Sequencing, clonal line testing
CRISPR_ext_F	GCCACATGTTGGAGACACTG	Clonal line testing
CRISPR_ext_R	ACAGTCTCACCTCGCCTTGT	Clonal line testing
CRISPR_int_F	AGTCTTTCACGCTGCGAAGT	Sequencing, clonal line testing
CRISPR_int_R	TGTATGCCGCTAGAGTGCTG	Sequencing, clonal line testing

When applicable, the restriction endocuclease (RE) is identified in the Primer ID.The sequence targeted by the RE is underlined. The nucleotides upstream of these were added to increase the efficiency of the restriction activity on the amplicons. [1]The first "G" was manually added to the 5’ of the gRNA target sequence.
