# Supplementary material for: Characterization of a MOB1 Homolog in the Apicomplexan Parasite Toxoplasma gondii
Source: Biology (Basel). 2021 Nov 26;10(12):1233. doi: 10.3390/biology10121233 (PMC8698288; doi:10.3390/biology10121233)
Supplement: Supplementary file 1 [file biology-10-01233-s001.zip › biology-1463695-supplementary/Table S3.pdf]

Table S3. Antibodies used for this work

Antibody	Species	Dilution	Source	Reference
**Immunofluorescence**				
Antibody	Species	Dilution	Source	Reference
polyclonal anti-T. gondii MOB1	mouse	1:100	in house	_
polyclonal anti-T. gondii TBCB	rabbit	1:100	in house	_
polyclonal anti-T. gondii surface proteins	rabbit	1:2000	in house	_
anti-IMC1	rabbit	1:500	Dominique Soldati-Favre	_
anti-FLAG	mouse	1:200	Sigma	F3165
anti-polyglutamylation modification GT335	mouse	1:200	Adipogen	ALX-804-885
Alexa Fluor 594-conjudated anti-mouse IgG	goat	1:500	Invitrogen	A-11005
Alexa Fluor 594-conjudated anti-rabbit IgG	chicken	1:500	Invitrogen	A-21442
Alexa Fluor 488-conjudated anti-mouse IgG	goat	1:500	Invitrogen	A-11001
Alexa Fluor 488-conjudated anti-rabbit IgG	donkey	1:500	Invitrogen	A-21206
**Western blot**				
Antibody	Species	Dilution	Source	Reference
polyclonal anti-T. gondii MOB1	mouse	1:1000	in house	_
polyclonal anti-B. besnoiti PDI	rabbit	1:5000	in house	_
anti-FLAG	mouse	1:2000	Sigma	F3165
anti-acetylated tubulin	mouse	1:2000	Sigma	T6793
HRP-conjugated anti-rabbit IgG	goat	1:1000	Invitrogen	G-21234
HRP-conjugated anti-mouse IgG	goat	1:5000	Jackson ImmunoResearch	115-035-003
